# Microbial Exudates as Biostimulants: Role in Plant Growth Promotion and Stress Mitigation

**DOI:** 10.3390/jox13040037

**Published:** 2023-10-01

**Authors:** Mariya Ansari, B. Megala Devi, Ankita Sarkar, Anirudha Chattopadhyay, Lovkush Satnami, Pooraniammal Balu, Manoj Choudhary, Muhammad Adnan Shahid, A. Abdul Kader Jailani

**Affiliations:** 1 Department of Mycology and Plant Pathology, Banaras Hindu University, Varanasi 221005, Uttar Pradesh, India; mariyaansari610@gmail.com (M.A.); ankitasarkar@bhu.ac.in (A.S.); lovkushsatnami5@gmail.com (L.S.); 2 Department of Environmental Biotechnology, Bharathidasan University, Tiruchirappalli 620024, Tamil Nadu, India; megalabiotech@gmail.com; 3 Pulses Research Station, S.D. Agricultural University, Sardarkrushinagar 385506, Gujarat, India; anirudhbhu@sdau.edu.in; 4 Department of Biotechnology, Sastra Deemed University, Thanjavur 613401, Tamil Nadu, India; pooranimamicrokk@gmail.com; 5 Plant Pathology Department, University of Florida, Gainesville, FL 32611, USA; m.choudhary@ufl.edu; 6 Horticultural Science Department, North Florida Research and Education Center, University of Florida/IFAS, Quincy, FL 32351, USA; mshahid@ufl.edu; 7 Plant Pathology Department, North Florida Research and Education Center, University of Florida, Quincy, FL 32351, USA

**Keywords:** microbial exudates, biostimulant, plant growth, plant defense, biotic stress, abiotic stress, plant microbiome

## Abstract

Microbes hold immense potential, based on the fact that they are widely acknowledged for their role in mitigating the detrimental impacts of chemical fertilizers and pesticides, which were extensively employed during the Green Revolution era. The consequence of this extensive use has been the degradation of agricultural land, soil health and fertility deterioration, and a decline in crop quality. Despite the existence of environmentally friendly and sustainable alternatives, microbial bioinoculants encounter numerous challenges in real-world agricultural settings. These challenges include harsh environmental conditions like unfavorable soil pH, temperature extremes, and nutrient imbalances, as well as stiff competition with native microbial species and host plant specificity. Moreover, obstacles spanning from large-scale production to commercialization persist. Therefore, substantial efforts are underway to identify superior solutions that can foster a sustainable and eco-conscious agricultural system. In this context, attention has shifted towards the utilization of cell-free microbial exudates as opposed to traditional microbial inoculants. Microbial exudates refer to the diverse array of cellular metabolites secreted by microbial cells. These metabolites enclose a wide range of chemical compounds, including sugars, organic acids, amino acids, peptides, siderophores, volatiles, and more. The composition and function of these compounds in exudates can vary considerably, depending on the specific microbial strains and prevailing environmental conditions. Remarkably, they possess the capability to modulate and influence various plant physiological processes, thereby inducing tolerance to both biotic and abiotic stresses. Furthermore, these exudates facilitate plant growth and aid in the remediation of environmental pollutants such as chemicals and heavy metals in agroecosystems. Much like live microbes, when applied, these exudates actively participate in the phyllosphere and rhizosphere, engaging in continuous interactions with plants and plant-associated microbes. Consequently, they play a pivotal role in reshaping the microbiome. The biostimulant properties exhibited by these exudates position them as promising biological components for fostering cleaner and more sustainable agricultural systems.

## 1. Introduction

The ecology of the phytosphere is highly complex, where continuous interactions between plants and microbes are evident. This interplay can either benefit each other through various mechanisms, such as nutrient exchange, the induction of metabolic pathways/processes, and the secretion of various exudates or metabolites, or harm each other through disease initiation, a reduction in plant growth, and the modification of the microbiome. Microbe–microbe interactions further shape the microbiome structure, including both beneficial and harmful microbes. These multi-trophic interactions create a unique chemical niche around the plant, determining the fate of phytospheric health and, ultimately, maintaining ecological balance in agroecosystems. Unfortunately, the introduction of xenobiotic compounds (organophosphates, aromatic hydrocarbons, heavy metals, and phenols) into agroecosystems in the form of pesticides, herbicides, and fertilizers has resulted in soil degradation and environmental deterioration.

Exploring and harnessing beneficial microbes is an emerging option forsolving this problem. These beneficial microbes include plant-growth-promoting rhizobacteria (PGPRs), plant-growth-promoting fungi (PGPFs), endophytes, biocontrol agents, mycorrhiza, algae, etc. They play multifunctional roles in direct and indirect plant growth and development, as well as promotion. Directly, they can interfere with other microbes through hyperparasitism, predation, and ecological competition. Indirectly, they can secrete various biologically active compounds such as sugars [1], enzymes, siderophores, 1-aminocyclopropane-1-carboxylate (ACC) deaminase [2,3], volatile organic compounds (VOCs) [4], exopolysaccharides [5,6,7], and phytohormones such as gibberellin, cytokinin, and abscisic acid [8,9,10]. These compounds act as biostimulants, regulating or modifying physiological processes in plants and mitigating stress conditions. Some of these compounds, like enzymes, can degrade xenobiotic compounds such as organophosphate pesticides through hydrolyzation [11], making them potential bioremediants. However, applying these beneficial microbes in consortia poses challenges in their large-scale (field) application due to their specific nutritional and environmental requirements for growth. Their high ecological specificity and preference for specific host plants and soil physio-chemical niches make them less suited for robust field application as they are well-adapted to their native environment. In addition, delivering beneficial microbes in the proper formulation in situ is crucial for building up their population, but a one-size-fits-all approach may not be suitable for all microbial species/strains. Furthermore, biosafety concerns related to the use of living micro-organisms may limit their direct field application.

A promising solution to address these challenges may arise from the rhizosphere, where plants and microbes exist as interacting entities, producing an array of diverse chemical compounds that play a crucial role in plant growth and development. Over 20,000 compounds produced by microbes can influence plants’ behavior, combat biotic and abiotic stress, and promote normal growth and development. Such compounds, collectively known as “biostimulants,” of microbial origin are included under the European Union (EU) regulation 2019/1009 as fertilizing products, providing they function to stimulate plant nutrition processes independent of products’ nutrient content and improve characteristics such as nutrient use efficiency, tolerance to stress, quality traits, or nutrient availability in soil, plants, or the rhizosphere [12]. In this context, microbial biostimulants consisting of microbial exudates play a significant role and are composed of diverse molecules such as sugars, organic acids, amino acids, peptides, siderophores, volatile compounds, etc. These exudates can easily be extracted from microbial cultures and applied as cell-free exudates. The CMC-7 (Component Material Categories, number 7) list includes microbes from only four different genera, like *Rhizobium* sp., *Mycorrhiza* sp., *Azotobacter* sp., and *Azospirillum* sp., and a microbial plant biostimulant can consist of these micro-organisms or their consortia. Such strict regulation could hinder and prohibit the use of novel beneficial microbes, as well as the formulation of their products in the market. Nevertheless, the European Biostimulant Industry Council (EBIC) has directed its focus to redefine its regulatory and safety requirement on the EU market and update the CMC-7 list in the new regulation [13] to encourage the inclusion of other beneficial microbes.

Currently, cell-free microbial exudates show great potential in various fields of agriculture, from stimulating plant growth and defense to the bioremediation of harmful pesticides and heavy metals. Due to their low environmental impact, these exudates can be preferred over fertilizers and pesticides, making them viable components for sustainable agricultural systems. However, since the precise definition of microbial exudates remains uncertain, this review aims to encompass and classify the diverse nature of compounds secreted or exudated by microbes, which can be referred to as microbial exudates. The review primarily focuses on the role of microbial exudates as biostimulants, and their function in plant growth, health promotion, plant protection, and the alleviation of abiotic stress. Additionally, the strategies or mechanisms involved in the cell-free microbial exudate-mediated remediation of heavy metals and the degradation of xenobiotic compounds are illustrated, along with their impact on the plant microbiome.

## 2. Microbial Exudates and Their Composition

**Figure 1:**
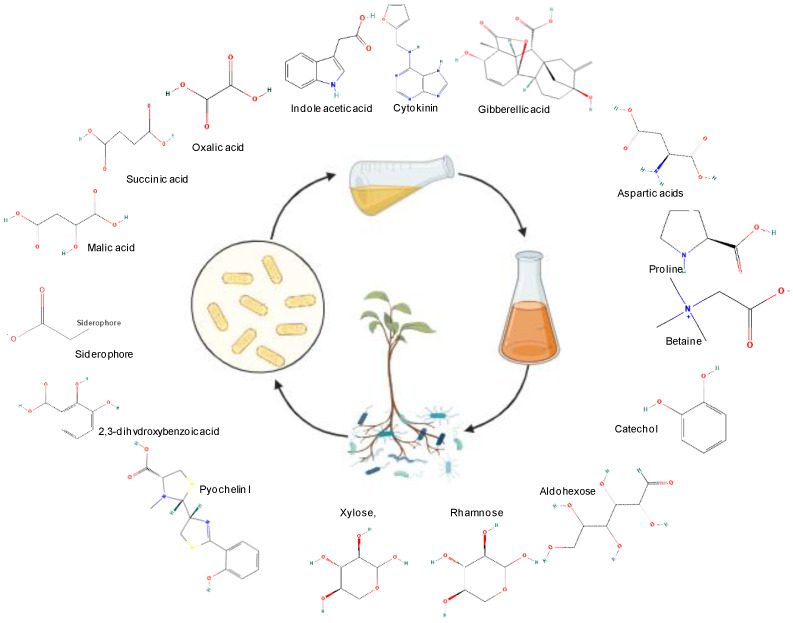
Biochemical constituents of microbial exudates containing amino acids (aspertic acid, proline, and betaine), exopolysaccharides (aldohexose, rhamnose, and xylose), siderophores (Pyochelin and 2,3 dihydroxybenzoic acid),organic acids (malic acid, succinic acid, and oxalic acid), hormones (auxin, cytokinin, and gibberellic acid), reducing agent (catechol), etc.

Microbes release a variety of exudates into the rhizosphere, where the term “exudates” refers to compounds released through exudation, secretion, or both. These compounds are diverse, with the majority being organic, inorganic, or chemical in nature. They include metabolites like hormones, organic acids, amino acids, exopolysaccharides, siderophores, hydrogen cyanide (HCN), and volatile organic compounds(VOCs) (Figure 1). Microbes produce these compounds in response to various stimuli, such as competition for niche species, nutrient deficiency, signals from plants, or as a response to environmental stress. Research indicates that, during the evolutionary phase, plants have developed sensing mechanisms to perceive a fraction of these compounds [14] to enhance their growth under adverse conditions [15,16,17].

### 2.1. Siderophores

Siderophores are low molecular weight organic compounds synthesized by numerous bacterial and fungal species under iron (Fe)-deficient environments. They are structurally and chemically diverse molecules with different iron-chelation capacities. Plants growing under iron-stressed conditions utilize siderophores produced by rhizospheric microbes surrounding the root zone to meet their iron demand. These siderophores have been reported to enhance biochemical and physiological processes in plants under drought conditions [18], saline soil [19], and heavy-metal-stressed soil [20]. Additionally, siderophores possess certain ligands to bind with free iron (chelation) and other metal ions such as molybednum (Mo^+6^), cobalt (Co^+2^), manganese (Mn^+2^), and nickel (Ni^+2^), and facilitate their transportation within the plant cell through membrane receptor molecules [21]. Siderophores are capable of oxidizing heavy metals such as lead (Pb^+2^), zinc (Zn^+2^), cadmium (Cd^+2^), uranium (U^+6^), plutonium (Pu^+4^), thorium (Th^+4^), and Ni^+2^, altering their toxicity [22]. Consequently, they play a crucial role in bioremediation, enhancing plant growth, and nutrient uptake [23]. Based on the ligands used for iron chelation, siderophores are divided into four major families, namely, catecholate, hydroxamate, carboxylate, and mixed-ligand siderophores.

#### 2.1.1. Catecholate Siderophores

These siderophores contain mono- or dihydroxybenzoic acid moieties engaged in the chelation of ferric iron via hydroxyl groups, forming a hexadentate octahedral siderophore–Fe^3+^complex when secreted. Various beneficial plant-associated bacterial species produce this type of siderophore, such as 2,3-dihydroxybenzoylglycine (*Bacillus subtilis*) [24], spirilobactin (*Azospirillum brasilense*), 2,3-dihydroxybenzoic acid (*Azospirillumlipoferum)* [25], aminocholine, nitrocholine, protochelin (*Azotobacter vinelandii*) [26], Agrobactin (*Rhizobium radiobactor*) [27], and 2,3-dihydroxybenzoic acid conjugated to threonine (*Rhizobium leguminosarum*) [28]. For example, the catecholate siderophore produced by *Bacillus subtilis* is reported to enhance seed germination and plant growth in *Coriandrum sativum* [29]. Under drought conditions, catecholate is reported to enhance soybean seed germination [30].

#### 2.1.2. Hydroxamate Siderophores

These siderophores are mostly derivatives of hydroxamic acids, with hexadentate ligands involved in the chelation of ferric (Fe^3+^) ions via a carbonyl group. They are produced by bacterial and fungal species and form stable and strong hexadentate octahedral complexes with Fe^3+^. Examples include Vicibactin (*Rhizobium leguminosarum* bv. *viciae*), Ferrichrome (*Ustilago ferrigona*), Desferridoxamine B (*Streptomyces griseus*), and ferribactin (*Pseudomonas fluorescens*). Hydroxamate produced by *Bacillus subtilis* MF497446 and *Pseudomonas korensis* MG209738 significantly increased polyphenol oxidase, catalase, and peroxidase activities in maize, along with boosting the plant chlorophyll and carotenoid content, leading to improved crop yield [31].

#### 2.1.3. Carboxylate and Mixed-Type Siderophores

Carboxylate siderophores are derivatives of citric acids, containing hydroxyl and carboxyl groups as proton donors for iron acquisition. They are produced by bacteria like *Sinorhizobium meliloti* (Rhizobactin) [32] and fungi like *Rhizopus microspores* (Rhizoferrin) and other mucorals [33]. Mixed-ligand siderophores encompass several functional groups for chelating iron, such as salicylic derivatives (pyovedine and pyochelin produced by *Pseudomonas* sp.) and hydroxymate and phenol catechol functional groups. Pyoverdine-like and Pyochelin-like siderophores produced by *Pantoea eucalypti* M91 under alkaline conditions were reported to promote morphological and biochemical changes in the lotus plant and induce improved photosynthesis and iron translocation [34].

### 2.2. Exopolysaccharides (EPSs)

Exopolysaccharides (EPSs) are extracellular sugar molecules produced by various microbes, including bacteria, cyanobacteria, marine microalgae, and marine micro-organisms [35]. They are secreted out of the cells and accumulate on the external surface of the synthesizing microbes. EPSs are composed of repeated units of the same or different types of sugar molecules coupled with proteins, glycoproteins, acids (glucuronic acid, mannuronic acid, or galacturonic acid), lipids (glycolipids), organic and inorganic compounds, metal ions, and extracellular DNA. As approximately 40–95% of the extracellular polymeric substance is composed of polysaccharides, EPSs are also used to designate compounds referred to as extracellular polymeric substances. EPS synthesis occurs via ATP binding cassette (ABC) transporter-dependent pathways, WZX/Wzy-dependent pathways, synthase-dependent pathways, or extracellular synthesis involving single sucrose proteins [36]. The resulting polysaccharides are modified by enzymatic reactions such as acylation, sulphation, methylation, and acetylation [37], and they are finally exuded from the cell in the form of loose slime or a capsule after being transported to the cell surface. EPSs are released into their surroundings in response to physiological stresses, such as temperature [38], salinity [39], or heavy metal pollution [40], to overcome extreme environmental fluctuations. The different composition (carboxyl, hydroxyl functional groups, and non-carbohydrate substituents) and structure of EPSs enable metal ion sequestration by biosorption through the interaction between positively charged metal ions and negatively charged EPSs [41]. EPSs are reported to act as a conductor and reservoir of water to plant roots under water stress [42], and they can chelate free Na+ from the soil, making it unavailable to plants under salt stress. While a comprehensive understanding of the direct impact of EPSs on plant physiology to improve drought stress is not absolute, Naseem and Bano [43] suggested that diverse functional groups of EPSs trigger different plant antioxidant mechanisms to alleviate drought stress. The flocculating property of EPSs allows the aggregation of primary soil particles, enabling the stabilization of the soil structure and the improvement of soil physical properties such as porosity and bulk density [44]. Due to these characteristics, EPS-producing PGPRs, when associated with plants, play a crucial role in alleviating abiotic stress and are widely used for bioremediation.

For example, Atouei et al. [5] reported that EPSs secreted by *Bacillus subtilis* TP7 and *Marinobacter lipolyticus* SM19 restricted the uptake of Na+ by wheat. The binding and biofilm formation property of EPSs favor soil aggregation, stability, and the retention of the water layer around root cells, improving cell adhesion in plants growing under salt stress [45,46] and drought stress [47,48]. Cheng et al. [49] demonstrated the effect of EPS-producing bacteria (*Pseudomonas chlororaphis* A20 and *Bacillus proteolyticus* A27) on the cell count, polysaccharide content, and invertase activity in the soil. They reported an increase in the cell biomass, polysaccharide content (by 158–174%), invertase activity (153–198%), and the ratio of water-stable soil macroaggregates and water-stable macro-aggregates in soil compared to the un-inoculated control, possibly due to the production of specific EPSs (xylose, aldohexose, rhamnose, and glucose). Furthermore, EPSs have been reported to slow down the evaporation process, increasing water availability in plants [50]. EPSs produced by the *B. subtilis* strain UD1022 reduced the hydraulic conductivity and accumulative evaporation in treated soil by altering/modifying water’s physiochemical properties (viscosity and surface tension), soil matrix structure, and pore space connectivity. This provides more time for plants to make necessary metabolic adjustments during drought stress. Benard et al. [51] reported a similar effect while working on the *B. subtilis* strain NCIB 3610, where EPSs produced by the strain reduced evaporative drying and water loss from the soil by reducing the soil hydraulic conductivity and capillary forces, enabling the retention of a water layer below the dry soil.

### 2.3. Phytohormones

Phytohormones like auxin, gibberellin, cytokinin, and ethylene are exogenously produced by diverse microbes, including rhizospheric, epiphytic, symbiotic, and pathogenic fungi and bacteria. These phytohormones act as mediators for communication between plant hosts and microbes and serve as natural growth promoters for plants. Indole acetic acid (IAA) belongs to indole derivatives and is produced by many rhizospheric and epiphytic bacteria, as well as methylobacteria. Its biosynthesis in micro-organisms is associated with tryptophan metabolism and is formed via indole-3-pyruvic acid, indole-3-acetic aldehyde, and indole-3-acetamide formation. In plants, IAA binds to amino acids, sugars, or proteins and is stored in an inactive form; it is released when required. Gibberellins, on the other hand, are complex molecules of tetra-carbocyclic diterpenes consisting of isoprene residues that form four rings (A, B, C, and D). The best-studied GAs are GA3, GA7, GA1, and GA4, and several other gibberellins are characterized based on characteristic biological activity. GAs in plants are required for stem elongation, cell division, the activation of amylolytic enzymes, and membrane stabilization. Another phytohormone, cytokinin, is produced by some microbes, such as rhizobacteria, streptomycetes, methylotrophic and methanotrophic bacteria, and PGPR strains, and includes adenine derivatives. These microbes synthesize zeatin, kinetin, iso-pentenyl-adenine, and some other cytokinin derivatives. Cytokinin regulates a wide range of physiological responses in plants, such as the activation of cellular RNA/protein synthesis, the inhibition of quiescence, the regulation of chloroplast formation, and the stabilization of the photosynthetic apparatus under adverse environmental stress. Additionally, certain bacteria and fungi can produce ethylene, which is biosynthesized via methionine metabolism. Phytohormones produced by microbes thus work as regulators for numerous physiological processes in both plants and microbes, can serve as a nutrient source, and have antimicrobial properties, as well as have a direct influence on microbial communities. Microbial auxins are reported to enhance root growth and root hair formation. Their capacity to sustain plant growth under abiotic stress or nutrient deficiency can be attributed to their role in altering root development and architecture [52]. Furthermore, microbial cytokinin can boost the release of root exudates (amino acids) in its rhizosphere, which may have a broader effect on rhizospheric microbiomes [53].

### 2.4. Volatile Organic Compounds (VOCs)

Microbial VOCs are a group of compounds emitted by microbes, such as bacteria and fungi, under diverse ecosystems. Chemically, microbial VOCs comprise heterogeneous molecular classes such as alcohols, ketones, thioalcohols, hydrocarbons, aldehydes, thioesters, cyclohexane, phenols, and benzene derivatives [36,54,55,56]. These compounds originate from catabolic backgrounds and encompass low-complexity, rather lipophilic compounds [57,58,59,60]. They include inorganic molecules (CO, H_2_, CO_2_, N_2_, NH_3_, SO_2_, SO_3_, H_2_S, O_2_, NO_2_^−^, and HCN) or organic molecules that are small (<300 Da) C-based molecules. Classification has also been performed based on their molecular features, such as ring moieties, the number of C-atoms, and substituent groups [61]. Microbial VOCs have immense functional potential, and, although their role in promoting plant growth is underestimated, it was first reported by Ryu et al. [62]. Microbial VOCs have been utilized to control pathogenic fungi in fruits and vegetables, and, although the exact molecular and physiological mechanisms behind it are not elucidated, the underlying mechanism includes the disruption of the fungal cell wall (by increasing membrane permeability via lipid peroxidation) and membrane structure leading to intracellular lysate leakage, and the induction of oxidative stress. For example, VOCs have been reported to increase cell membrane fluidity, leading to conformational changes in membrane proteins, the leakage of intracellular content and eventually, the death of the fungal cell [63].

### 2.5. Organic Acids and Amino Acids

Distinguishing between microbial exudates and plant root exudates is challenging in an actual plant–microbe interacting set-up, as both are interrelated and collegial. Many researchers use the term “exudates” for compounds released by roots and associated microbes [64]. Exudates contain different types of compounds like organic acids (oxalic acids, citric acids, malic acid, succinic acid, etc.), reducing agents (catechol, phenolic substances, etc.), and simple sugars [64] and amino acids (aspartic acid, etc.) [65]. These compounds have different influences on the rhizospheric microbiome. Simple sugars act as easily digestible food for microbes, attracting more microbes to the rhizosphere [66]. As a result, there is a higher formation and loss of mineral-associated organic matter (MAOC) [65]. MAOC is the organic matter complexed with amorphous iron (Fe) and aluminum (Al) oxides, mostly a byproduct of microbial biomass [64]. However, if the exudate is rich in amino acids, there is a net accumulation of mineral-associated organic matter (MAOM) [65]. On the other hand, when there is more mineralization of MAOC, it also makes the reservoir of fixed nitrogen available for plants and microbes [67]. Amino acid arginine was detected in the exudates of the biofilm of *Pantoea* sp. on the roots of poplar, and its concentration was positively correlated with biofilm formation [68]. Oxalic acid greatly contributes to the mineralization of MAOM and also positively regulates the population of alkaline phosphatase gene-harboring microbes like *Pseudomonas* and *Bacillus*, as well as the phosphorus concentration of rhizospheric soils [69]. Organic acids and reducing agents serve as stronger agents carrying out the mineralization of MAOM, which is complexed with reducible forms of ferric oxides. As a result, they release the ferric or ferrous ions from the complex. Plant-growth-promoting microbes release carbon and nitrogen from ferric complexes to make nutrients more available to plants and microbes, and, to prevent further capturing of these nutrients by the ferric ions, the microbes further chelate them by virtue of producing siderophores. Several reports also claim that this chelating of iron creates iron-deficient conditions in the rhizospheric region of plants, which activates the defense-related induced systemic resistance pathway in plants [70].

## 3. Identification and Characterization of Microbial Biostimulants

Microbial biostimulants are essential for promoting plant growth and stress resistance. However, the emphasis has recently changed to employing cell-free microbial exudates as biostimulants to tackle the problems of shelf life of microbial cells and spores. To find secondary metabolites in microbial exudates, new methods have been devised [71]. The challenging objective is to identify the precise components and compounds that, when applied as biostimulants, have a positive influence on plant growth and stress resistance. Biochemical profiling (co-cultivation and chemical epigenetic manipulation), molecular identification (transcriptional regulation and promoter tools), biological assays (cultural conditions), and other techniques can be used to determine the biochemical nature of biostimulants in microbial exudates [72,73] (Table 1).

Before beginning these analyses, microbes are grown in liquid broth media, followed by refrigerated centrifugation, collection of the supernatant, vigorous shaking, and separation using a separating funnel. This procedure extracts microbial exudates using ethyl acetate, ethanol, or methanol. In order to discover new secondary metabolites as biostimulants, the resulting fraction is further separated and gathered for a biological assay and biochemical investigation [74].

### 3.1. Biological Assays

#### 3.1.1. In Vitro Study

Separate fractions can be dried and dissolved in sterilized distilled water or methanol. A methylthiazolyldiphenyl-tetrazolium bromide (MTT)-based assay in microtitre plates can be used to determine the optimal dose for increasing the germination percentage without having any phytotoxic effects [75]. The fractions can be added to Murashige and Skoog (MS) medium in a variety of concentrations, and observations of various plant seedling parameters like germination rates, root growth, shoot development, fresh weight, and dry weight can be made [76]. This approach is known as the multi-trait high-throughput screening of plants (MTHTS). It is also possible to assess how the plants react to abiotic challenges including salt, dehydration, and cold tolerance under the influence of the fractions [77].

#### 3.1.2. In-Pot Assay

As foliar sprays for dipping roots prior to transplanting or for irrigating seedlings placed in sterilized soil, the stated water and organic fractions can be utilized. The impact on seedling biomass accumulation, root shape, shoot biomass index, yield, and nutritional status can be evaluated [78,79]. The state of defense and phytohormonal signaling enzymes and molecules can be evaluated at the transcriptome level under biotic and abiotic stress conditions [80]. The impact of biostimulants on the methylation state of the plant genome can also be investigated [81].

#### 3.1.3. On-Field and Hydroponics Study

The specified doses of biostimulants can be evaluated through hydroponics and field trials, followed by field crop phenotyping through drone imaging [82]. To assess the effect on crops, integrating analysis with different omics approaches and advanced statistical tools is needed [83]. The potential of microbial exudates can be identified and chemically deciphered.

### 3.2. Biochemical Assay

**Figure 2:**
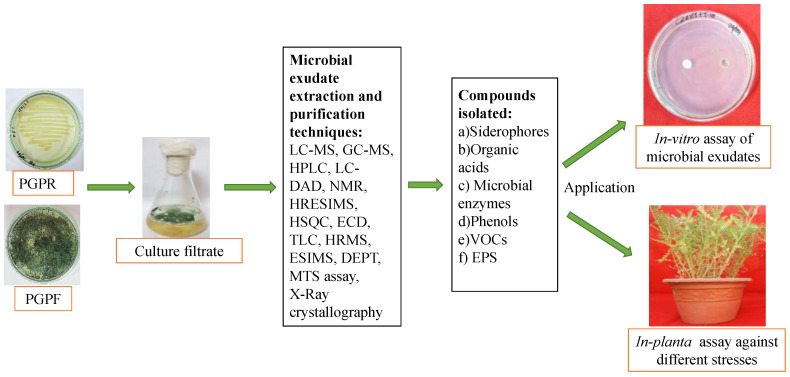
Isolation, purification, and characterization of microbial exudate and its application. Pure culture of microbes, viz., plant-growth-promoting rhizobacteria (PGPRs) and plant-growth-promoting fungi (PGPFs) were grown in suitable liquid culture media. Then, the cell-free microbial culture filtrates can be characterized by advanced techniques like liquid chromatography–mass spectrometry (LC-MS), gas chromatography–mass spectrometry (GC-MS), high-performance liquid chromatography (HPLC), liquid chromatography–diode array detection (LC-DAD), nuclear magnetic resonance(NMR) spectroscopy, high-resolution electrospray ionization mass spectrometry (HR-ESI-MS), heteronuclear single quantum coherence (HSQC) spectroscopy, electronic circular dichroism (ECD), thin layer chromatography (TLC), high-resolution mass spectrometry (HRMS), electrospary ionization mass spectrometry (ESI-MS), distortionless enhancement by polarization transfer (DEPT), MTS assay, X-ray crystallography, etc. The culture filtrates containing siderophores, organic acids, microbial enzymes, phenols, VOCs, EPS, etc. were isolated, identified, and further tested through in vitro and in planta assay.

Different analytical methods, including thin layer chromatography (TLC), gas chromatography coupled with mass spectrometry (GC-MS), liquid chromatography coupled with mass spectrometry (LC-MS), high-performance liquid chromatography (HPLC), column chromatography (CC), and high-resolution mass spectrometry (HRMS), etc. can be used to examine the fractions that have positive effects on plant growth and stress resilience [74,84,85,86]. The dereplication of samples and analysis of microbial exudates using advanced tools like high-performance liquid chromatography (UHPLC)-diode array (DAD)-HRMS and databases like NIST, Global Natural Product Social (GNPS) Molecular Networking platform, and Dictionary of Natural Products Database (DNPD) can help identify novel secondary metabolites from microbes (Figure 2) [74,87].

### 3.3. Molecular Identification

If the microbe’s entire genome sequence is available, or once it has been generated, tools like the antiSMASH and KEGG pathway analyser can be used to undertake genome annotation and secondary metabolite gene cluster analyses [88,89].This information can be used to validate the gene function through gene silencing or editing techniques [90].

The molecular identification of genes associated with biostimulant production in microbes is essential for their further exploitation. The medium can be modified to increase the production of the desired metabolite once it has been identified as a biostimulant in the microbial exudate. Alternatively, mutants can be generated to increase the metabolite’s production. Genes responsible for metabolite synthesis can be engineered and introduced into *E. coli* for low-cost commercial production, which is crucial for agricultural crop productivity and stress resilience [91].

**Table 1:** Some techniques for detection and identification of microbial exudates.

Microbe	Detection Techniques	Compounds Detected	Property of Compound	Reference
*Trichoderma harzianum*	OSMAC, extraction with ethyl acetate, LC-MS, GC-MS, X-ray analysis, plant growth, antifungal assay, cytotoxicity assay.	Siderophores, (ferricrocin and coprogen B), harzianic acid (HA) and its derivatives, butenolides and a novel metabolite, 5-hydroxy-2, 3-dimethyl-7-methoxychromone	Antifungal, anticancerous, no cytotoxic effect	[92]
*Alcaligenes faecalis*	Co-cultivation with fragments of *Sclerotium rolfsii*, extraction by ethyl acetate, HPLC, poisoned food technique, in-plant assay of defence and growth promotion.	Higher concentration of shikimic acid and gallic acid in CFS during co-cultivation. Higher concentration of defence enzymes in plants challenged and sprayed with CFS of co-cultivated A. faecalis.	Antifungal, plant growth promoter, and plant defense promoter	[72]
Actinomycetes (*Micromonospora* sp. UR56 and *Actinokinespora* sp. EG49)	Co-cultivation with Actinomycetes or other non-actinomycete bacteria, fungi, cell-derived components, and/or algae.OSMAC.	1,6-Dicarboxylate	Antibacterial	[93]
		Carbazoquinocin G	Antimicrobial	[94]
		Malformin C	Increase in cytotoxic activity	[95]
*Trichoderma* spp.	α,α-diphenyl-β- picrylhydrazyl (DPPH) free radical assay for total phenolic, ascorbic acid, total antioxidant capacity, anthocyanin characterization, fruit protein analysis by bioinformatics and Nano LC-ESI-Q-Orbitrap MS/MS.	6-pentyl-α-pyrone (6PP), harzianic acid (HA), and hydrophobin 1 (HYTLO1)	Growth promotion of strawberry, more synthesis of proteins, activated defense response in plants after treatment with specified compounds	[96]
*Trichoderma brevicompactum*	Preparative TLC, NMR, HR-ESI-MS, X-ray crystallography	Trichodermarins G–N, trichodermol, trichodermin, trichoderminol, trichodermarins A and B, 2,4,12-trihydroxy apotrichothecene	Antifungal and antimicroalgal activities	[97]
*T. brevicompactum* TPU199	Fermentation with sodium halides, LC-MS, NMR	Trichobreols A–C	Antifungal activity	[98]
*T. longibrachiatum*	Extraction with ethyl acetate, silica gel vaccum liquid chromatography, HPLC, HR-ESI-MS, NMR, HSQC, ECD spectra, microdilution.	Trichothecinol A, 8-deoxy-trichothecin, trichothecinol B, Trichodermene A	Antifungal activity	[99]
*T. atroviride* B7	Extraction with ethyl acetate, TLC, HPLC, CC, preparative TLC, semi-preparative HPLC, NMR. HRMS, COSY, key HMBC and key ROESY correlation of compounds, MTS assay for cytotoxicity	Harzianols F–J, 3S-hydroxyharzianone, harziandione, harzianol A	Potent antibacterial activity and moderate cytotoxicity	[100]
*T. virens* FKI-7573	Molecular identification, MS, NMR, ECD, and chemical degradation and comparison with DNPD.	Trichothioneic acid	Potent antioxidant activity	[101]
*T. afroharzianum* Fes1712	Overexpression of talae1, insertion of transformant plasmids (nested PCR and vector-based strategy) of *E.coli* into *T. afroharzianum* Fes1712 for secondary metabolite production. Ethly acetate extraction, CC, semi preparative HPLC, HRMS, NMR, ECD, bioactivity (96-well titer plate microdilution).	(R,3E,5E)-1-(3,5-dihydroxy-2,4- dimethylphenyl)-1-hydroxyhepta-3,5-dien-2-one, (R,3E,5E)-1-(3,5-dihydroxy-2,4- dimethylphenyl)-1-methoxyhepta-3,5-dien-2-one	Moderate antifungal activity	[91]
*T. harzianum* QTYC77	Ethyl acetate extraction, NMR, HRMS, COSY spectra, HMBC spectra, HMQC spectra, DEPT spectra, UV spectra, CD spectra, IR spectra, UHPLC-QTOF-MS	Azaphilones D and E	Moderate antibacterial activity	[102]
*T. harzianum* D13	Ethyl acetate filtrate, ECDspectra, spectrophotometer, The 1D (1H, 13C, and NOE) and 2D NMR spectra [HMQC, (COSY), (HMBC), and (NOESY)], ECD spectra, ESI-MS, and HRESIMS, HPLC, CC, 96-well microtitre plate assay for antifungal activity.	Nafuredin C, nafuredin A	Moderate antifungal activity	[103]
*T. asperellum* IRAN 3062C and *T. longibrachiatum* IRAN 3067C w	Co-cultivation, methanol/ethanol extraction, reverse-phase HPLC, ESI-MS, RNA-extraction-based expression of *tex1* peptaibol synthetase gene.	Increased expression of *tex1* peptaibol synthetase gene and increased synthesis of Peptiabol when co-cultivated with plant pathogens	Antifungal activity	[73]

Abbrebiation: OSMAC = one strain, many compounds; ECD = electronic circular dichroism; ROESY = rotating frame Overhauser enhancement spectroscopy; HMQC spectra = heteronuclear multiple quantum correlation; DEPT = distortionlessenhancement by polarization transfer; NOESY = nuclear Overhauser effect spectroscopy;LC-MS = liquid chromatography–mass spectrometry; GC-MS = gas chromatography–mass spectrometry; HPLC = high-performance liquid chromatography;LC-DAD = liquid chromatography–diode array detection; NMR = nuclear magnetic resonancespectroscopy; HR-ESI-MS = high-resolution electrospray ionization mass spectrometry; HSQC = heteronuclear single quantum coherence spectroscopy; ECD = electronic circular dichroism; TLC = thin layer chromatography, HRMS = high-resolution mass spectrometry; HSQC = heteronuclear single quantum correlation NMR spectroscopy;UHPLC-QTOF-MS = ultra-high-performance liquid chromatography–quadrupole time-of-flight–mass spectrometry.

## 4. Microbial Exudates as Biostimulants

Biostimulants encompass natural, synthetic, or formulated products of biological origin that can modify or regulate plant physiological processes, ultimately improving plant health and growth. The majority of biostimulants are of microbial origin and consist of secretions, extracts, or exudates from various microbes, including bacteria (endosymbiotic and plant-growth-promoting bacteria), fungi (mycorrhizal or non-mycorrhizal fungi), and algae. Endophytic microbes produce metabolites with diverse biological activities, such as alkaloids, polypeptides, polyketides, and terpenoids, which hold significant importance in various fields, particularly agriculture. Antimicrobial compounds and phytohormones released by endophytes play a crucial role in enhancing biotic stress tolerance and promoting plant development and growth. These microbial exudates function through direct and indirect mechanisms, facilitating plant growth promotion and regulating plant defense against biotic and abiotic stress [104,105]. While the exact mechanisms are not entirely understood, some researchers have illustrated different modes of action for these compounds. Certain microbial compounds act as signaling molecules, regulating defense gene expression, and phytohormone, phenol, or secondary metabolite synthesis in plants [106,107], and enhancing the production of enzymes or proteins essential for stress management [108,109]. The effectiveness of these compounds is influenced by factors like the nature of plant–microbe interactions, environmental conditions, and the type or concentration of compounds [17,110]. Currently, many microbes and their secreted compounds are well-characterized [111,112] and utilized for commercial biostimulant formulations.

### 4.1. Microbial Exudates in Promoting Plant Growth and Health

Microbial exudates can promote and stimulate crop growth and development through various mechanisms, such as the solubilization of insoluble minerals, production of organic acids, antimicrobial metabolites/lytic enzymes, or regulation of growth-regulating genes (Table 2). These mechanisms can alter plant morphology, leading to increased root and shoot length, higher chlorophyll content, an expanded leaf area, extended flowering periods, and improved yields [113,114,115]. For instance, certain plant-growth-promoting microbes (PGPMs) enhance the nutrient utilization of associated plants by secreting organic acids and enzymes in the soil, facilitating the solubilization of potassium and inorganic phosphates. They also promote phosphorus mineralization through enzymes like phytases and acid phosphatases [116,117,118]. Additionally, ACC-deaminase produced by specific bacteria elevates stress hormone levels like jasmonic acid and salicylic acid, inducing plant defense by regulating key signaling pathways [119,120,121,122]. Some bacteria producing HCN increase the sequestration of metals and make phosphorus more available to their plant host [123]. Moreover, hormones produced by PGPMs increase root biomass, reduce stomata density and dimensions, and activate auxin-responsive genes, enhancing plant growth and development [114,124]. Notably, IAA produced by beneficial microbes stimulates lateral root formation and root surface area expansion, leading to improved nutrient uptake and plant growth [125,126]. Additionally, fungal siderophores secreted by species like *Aspergillus niger*, *Trichoderma harzianum*, and *Penicillium citrinum* promote shoot and root length in chickpeas [127]. Microbial cellular exudates also contain signature molecules that induce plant growth and defense; certain bacteria and algal species demonstrate these effects [128]. For instance, cell exudates of *Bacillus pumilus* and *Pseudomonas pseudolcaligenes* stimulate rice growth and yield [129]. Algal extracts, rich in osmolytes (proline and glycine betaine) and plant hormones (auxin, gibberellins, cytokinin, indole butyric acid, polyamine, and trans-zeatin), exert beneficial effects by activating plant growth and defending against biotic and abiotic stress.

### 4.2. Microbial Exudates in Alleviating Biotic and Abiotic Stress

In addition to promoting plant health, microbial exudates induce or stimulate the plant defense system against diverse biotic and abiotic stresses through modifications in physiological, biochemical, and biological properties (Table 3).

#### 4.2.1. Microbial Exudate as Plant Protectants

**Figure 3:**
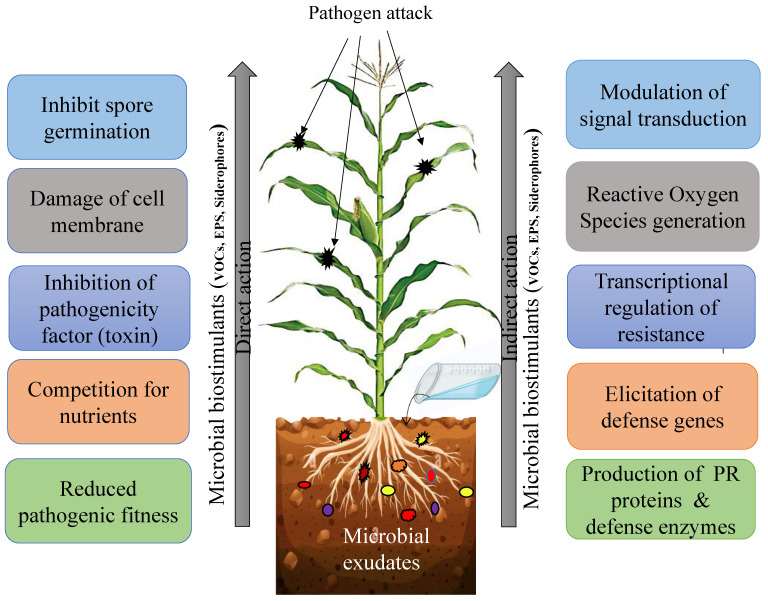
Induction of biotic stress tolerance through microbial exudates. Application of microbial biostimulants, viz., exopolysaccharides, siderophores, and volatile compounds directly and indirectly protect the plants from diverse pest and pathogen attack. Directly, they hamper the activity of the pathogen through restricted spore germination, damage of cell membrane, inhibition of pathogenicity factors, competition for nutrients, and reduced pathogenic fitness that, in turn, affect the pathogenicity and survivability of pathogens. Microbial biostimulants can induce the plant defense system through modulation of signal transduction pathways, generation of reactive oxygen species (ROS), transcriptional regulation of resistant genes, elicitation of PR protein synthesis, as well as production of secondary metabolites, etc.; thus, providing overall protection for the plant.

Microbial exudates contain several active compounds, such as hormones, exopolysaccharides, and volatiles, capable of inducing plant defense against various pathogens (Table 3). These compounds serve as elicitors of plant defense responses and activate different signaling pathways, such as salicylic acid, jasmonic acid, and ethylene (Figure 3). Hormonal signals target transcription factors (TFs) to regulate various genes and activate multiple plant metabolic pathways [161]. The priming effect of microbial exudates on the plant basal immune system confers broad-spectrum resistance against pathogens, effectively inhibiting biotrophic phytopathogens, including plant viruses, as well as hemibiotrophic and necrotrophic pathogens, such as *Fusarium* sp., *Sclerotinia* sp., *Rhizoctonia* sp., *Alternaria* sp., *Pythium* sp., and *Phytophthora* sp. [162,163,164,165,166,167,168]. Certain toxins, enzymes, or proteins secreted by microbes such as bacteria, virus, fungi, or microsporidia have pesticidal properties and are used to destroy and prevent the growth of pests. Certain secondary metabolites and protease (Serine protease 1) released by *Bacillus firmus* (I-1582) arereported to be effective against various plant parasitic nematodes [169,170]. Similarly, *Brevibacillus laterosporus* (UNISS 18) is reported to secrete certain enzymes like chitinase (chiA, chiD), bacillolysin (Bl18), collagenase-like protease (prtC), and insecticidal toxin (mtx) capable of targeting wide range of Dipterans, Coleopterans, Lepidopterans, and nematodes [171,172,173]. Shehata et al. [174] reported the presence of seven bioactive compounds [(hexadecanoic acid methyl ester (7.6%), phenol, 6-octadecenoic acid methyl ester (26%), pentadecane (4.1%), 2-methyldecane (1.3%), and Dotriacontane (2.5%)] in the concentrated cell-free supernatant of lactic acid bacteria (LABs) is active against *Fusarium* sp. VOCs produced by *P. fluorescens* ZX, mainly organic acids and sulfur compounds, significantly inhibiting the conidial germination and mycelial growth of *Penicillium italicum*, and reducing blue mold decay on postharvest citrus [175,176].

Exopolysaccharides produced by *Lactobacillus planetarium* elicit defense gene expression in tomatoes, increasing the catalytic activity of intracellular defense enzymes, such as PAL, PO, and polyphenoloxidase, and regulating the generation of reactive oxygen species (ROS) through catalase, superoxide dismutase, and hydrogen peroxide production [177]. The exogenous application of exopolysaccharides from *Pseudomonas fluorescence* LPK2 and *Sinorhizobium fredii* KCC5 induces the synthesis of chitinase and β-1, 3-glucanase in plant hosts, suppressing Fusarium wilt caused by *F. udum* and *F. oxysporum* [178]. Phenazine-1-carboxylic acid production by *P. fluorescence* LBUM223 negatively regulates the *txtA* gene (virulence and pathogenicity gene) expression of *Streptomyces* sp. and thaxtomin A production, resulting in the inhibition of Streptomyces spin potato infection [179]. Along with exopolysaccharides, siderophores, and microbial VOCs, microbial hormones and metabolites have the potential to enhance plant defense. The exogenous application of a metabolic cocktail (consisting of IAA, Indole-3-ethanol, SA, and indole-3-lactic acid) released from the microbial culture of *Azospirillum brasilense* strain V5 and V6 increases the expression level of defense genes like pathogenesis-related (PR) proteins and oxidative-stress-responsive genes in maize plants, enhancing plant growth [180,181]. Further, organic acids such as propionic, caproic, butyric, acetic, formic, and n-valeric acid produced by LAB strains are reported to have broad-spectrum activity against *Fusarium* sp. [182,183,184]. Similarly, the metabolic extract consisting of small, secreted cysteine proteins (SSCPs) produced by *Trichoderma virens* enhances the symbiotic relationship between plants and microbes and elicits the plant’s defense response to pathogens and parasites [185].

Under the European Green Deal policy, the ‘From Farm to Fork’ strategy aims to reduce the use of pesticides by 50% till 2030 to narrow down chemical interventions in agriculture. To address the policy’s target of a climate-friendly approach, this strategy will encourage the adoption of alternative pest and disease management practices and pave the way for microbial-based active compounds and products. Such microbes can be explored for use as plant protection products (PPPs).

#### 4.2.2. Alleviation of Abiotic Stress

**Figure 4:**
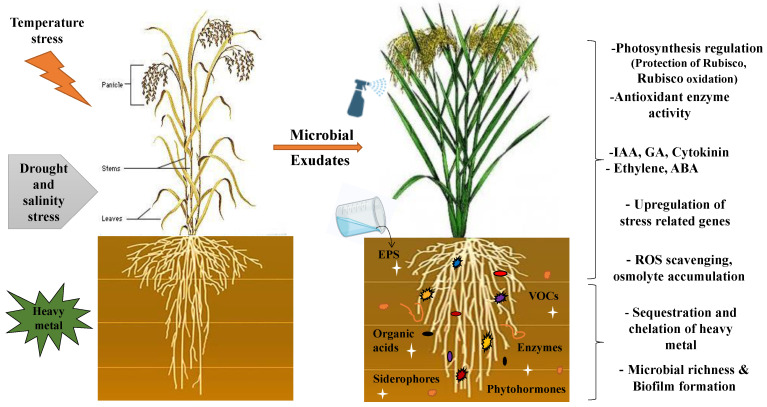
Alleviation of abiotic stress through microbial biostimulants like EPSs, phytohormones, siderophores, volatiles, etc. conferred through photosynthetic regulation, antioxidant production, phytohormone synthesis, upregulation of stress-related genes, elimination of heavy metals, and biofilm formation around root surface that leads to the morphological modification of plant to sustain various abiotic stresses.

The potential of microbial biostimulants to reprogram plant defense systems against abiotic stresses like heavy metal toxicity, osmotic stress, and heat stress is still not fully recognized. However, the remarkable ability of microbial biostimulants, such as exopolysaccharides, siderophores, and other compounds, to enhance abiotic stress tolerance in plants makes them a suitable choice for mitigating the adverse effects of climate change on crop physiology (Figure 4). Extracellular polymeric substances (EPSs) of microbe origin, including polysaccharides, glycoproteins, lipopolysaccharides, and peptides, can chelate, precipitate, and adsorb heavy metals by altering their mobilization (Table 3). For instance, microbial exopolymeric substances containing alginic, glucuronic acid, galacturonic acid, and uronic acid extracted from *Pseudomonas aeruginosa* and *Pseudomonas putida* influence the chromium bioavailability, solubility, and transport or sorption behavior in subsurface systems [186,187]. Under drought stress, EPSs secreted by *Bacillus amyloliquefaciens* FZB42 are reported to protect plants by enhancing the biofilm stability in *Arabidopsis thaliana* [7]. Additionally, EPSs can reduce Cr (VI) (highly toxic to all living organisms) to Cr (III), which has a lower solubility, less toxicity, and high sorptive characteristics [188,189]. This conversion can be an effective method for alleviating subsurface Cr (VI) contamination. Apart from EPSs, microbial siderophores (ferric iron chelating compounds) can detoxify heavy metals such as Cr^3+^, Al^3+^, Cu^2+^, Eu^3+^, and Pb^2+^ [23,190,191]. Siderophores released by *Azotobacter chroococcum* help in alleviating heavy metal stress in maize [192]. Similarly, siderophores secreted by *Agrobacterium radiobacter* were effective in removing 54% of arsenic from polluted sites [193]. Under salinity stress, the *P. citronellolis* strain SLP6 H is reported to enhance the chlorophyll content, production of antioxidant enzymes, and plant growth in *Helianthus annuus* by producing the Hydroxamate siderophore [154]. The application of siderophore-producing microbe *B. aryabhattai* MS3 in rice boosted crop production by 60% and 43% under non-saline and saline (200mM NaCl) conditions, respectively [19].

In this context, the exploration and utilization of plant-growth-promoting rhizobacteria (PGPRs) present a promising avenue. Some PGPRs alleviate drought stress in plants by producing certain VOCs, 1-aminocyclopropane-1-carboxylate (ACC) dismutase, EPSs, phytohormones, antioxidants, etc. The ACC-deaminase enzyme produced by PGPR strains can degrade ACC produced during environmental stress conditions, thereby reducing ethylene levels in the soil and improving plant health during drought [194]. Exopolysaccharides secreted by PGPR can mitigate heat stress in plants by promoting biofilm formation and encapsulating root nodules, improving the water retention capacity in plant roots. This, in turn, results in modifications in root surfaces and regulates osmolytes and stress-responsive genes [195,196,197], leading to the increased production of heat shock proteins [198].

Plants colonized with rhizobacteria can uptake proline produced by their bacterial partners with minimal or no modification [199]. This proline is taken up into the mitochondria using amino acid transporters and functions as an osmoprotectant, alleviating stress by preventing lipid peroxidation under metal stress, reducing ROS-mediated cell death, and efflux of K^+^ under salt stress. Proline can also act as a chemical chaperone to stabilize proteins [200], and it increases the leaf water potential during drought stress. Glycine betaine (GB) is synthesized by *B. subtilis* from its precursor glycine betaine choline by GbsB, GbsA, and GbsB enzymes [201]. Under high osmolarity, the uptake of glycine betaine choline is facilitated by five ABC transport uptake systems: OpuA, OpuB, OpuC, OpuD, and OpuE. GB plays a key role in maintaining photosynthetic efficiency by protecting Rubisco and Rubisco oxidase under stress conditions [202]. Additionally, under salt stress, GB can increase the accumulation of K^+^ ions or reduce Na^+^ ions in shoots [203,204]. Glomalin, a glycoprotein produced by *Glomus* sp., has been reported to have soil aggregating properties that improve the soil composition and provide drought tolerance [205]. In such a way, microbe-encoded complex compounds contribute to the amelioration of abiotic stress.

## 5. Microbial Exudates as Environmental Protectors

Xenobiotic compounds are manmade or chemical compounds introduced into the environment through industries, fossil fuel spills, mining activities, and agriculture, releasing excessive amounts of fertilizers, pesticides, and herbicides. These xenobiotics exhibit long-term persistence and slow degradation, leading to deleterious effects on the environment, soil, plants, and living organisms. Once they enter the food chain, they bioaccumulate, exerting carcinogenic, mutagenic, and toxic effects on organisms at higher trophic levels. Moreover, they alter the physio-chemical properties of the soil, microbial activity, and diversity, leading to ecotoxicological effects [206]. In plants, xenobiotics interfere with morphological and physiological characteristics, such as plant growth, seed germination, and changes in gene regulation and expression. They can also deregulate signaling pathways by interfering with signal receptors like G-Protein-coupled receptors and receptor tyrosine kinase [207]. Pesticides, fertilizers, and herbicides are major xenobiotic pollutants in agricultural systems, and they can bind with free metal ions in the soil to form complexes, reducing the bioavailability of essential nutrients for plants [208].

The hazardous impact of xenobiotics necessitates immediate degradation methods. Although physical and chemical degradation methods like adsorption, electrolysis, filtration, coagulation, ozonation, and chemical precipitation are available, their stringent, complicated, and high-cost methodologies, as well as toxic by-products, are major drawbacks for their application in xenobiotic degradation [209,210]. Alternatively, microbial-assisted degradation has emerged as the most appropriate, cost-effective, and environmentally friendly method over the past few decades. Xenobiotic-degrading fungi and bacteria have the metabolic ability to transform organic pollutants into less harmful compounds. They secrete a wide range of enzymes that enable them to utilize xenobiotics as their carbon and energy source. Several genes, enzymes, and degradation pathways are involved in biodegradation. Some primary microbial enzymes involved in biodegradation include laccase, cellulase, phytase, lipase, oxygenases, cytochrome P450s (mono-oxygenases), lignin peroxidase, esterase, and versatile peroxidases [206,211]. The main mechanisms for microbial xenobiotic degradation include reduction, oxidation, and hydrolysis [212,213]. Various multi-omics-approach studies on microbes have revealed specific genes encoding xenobiotic degradative enzymes, metabolites, and metabolic pathways of xenobiotic degradation. They have also elucidated differentially expressed catabolic genes under xenobiotic stress. For instance, functional metagenomics studies on *Koribacter*, *Acidomicrobium*, *Bradyrhizobium*, and *Burkholderia* revealed the abundance of phosphodiesterase-encoding genes, potentially capable of degrading organophosphorus compounds. These genera of bacteria were found in soil contaminated with pesticides [214]. Similarly, a transcriptomic study of DDT-resistant *Trichoderma hamatum* FBL 587 reported the upregulation of about 1706 genes involved in DDT degradation. Various DDT-metabolizing enzymes like epoxide hydrolases, glycosyl and glutathione-transferases, and FAD-dependent mono-oxygenases were also upregulated [215]. The degradation mechanism of the organophosphorus pesticide phoxim by *Bacillus amyloliquefaciens* YP6 was illustrated through transcriptome analysis, revealing the upregulation of oxidase, NADPH-cytochrome P450 reductase, and hydrolase genes for the oxidation, dealkylation, and hydrolysis of phoxim [216]. Microbial enzymes and their mechanisms involved in xenobiotic degradation, with special reference to pesticides, have been illustrated in Table 4.

## 6. Impact of Microbial Exudates on the Plant Microbiome

Microbial exudates enclose an array of molecules, including sugars, organic acids, hormones, secondary metabolites, polymers (mucilage), proteins, peptides, volatiles, and more [225]. While their effect on plant growth and stress tolerance is well-known, their impact on the wider plant microbiome remains abstruse. The production and release of exudates may not be essential for microbial growth and development, but they play a crucial role in shaping the microbial communities that coexist with them [226]. Although these exudates may not be required for the producers’ growth, they reduce niche competition within the microbial population, leading to a shift in microbial populations and promoting the proliferation of beneficial microbiota. Exogenously applied microbial exudates create a distinct chemical niche that favors the colonization of beneficial microbes in the phylloplane and rhizosphere, while deterring harmful microbes, thereby altering the microbial composition [227].

**Figure 5:**
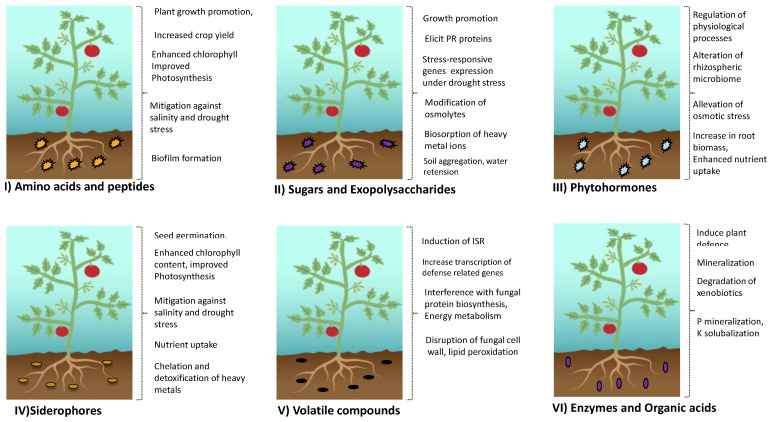
Effect of different components of cell-free microbial exudates on plant and its associated microbiome. Different components of cell-free microbial exudates, viz., amino acids, peptides, sugars, saccharides, exopolysaccharides, etc. lead to physiological and biological changes in the plant, as well as microbiome composition of the rhizosphere, through the encouragement of some specific beneficial microbes and reduction in the number of harmful microbes.

It can be presumed that chemically similar compounds are secreted by a group of microbes and their relatives that belong to the same taxonomic clade. Sometimes, these chemical exudates have either beneficial or harmful effects on other microbial populations. They may be used as metabolic substrates and growth regulators, or act as toxic, antagonistic compounds, and signaling molecules, ultimately altering the microenvironment and influencing the population of surrounding organisms. The composition and functioning of microbial exudates play a key role in shaping the plant microbiome (Figure 5).

### 6.1. Microbial Exudates as Food for Other Microbes

Most microbial exudates consist of a substantial amount of carbon-containing compounds such as sugars, organic acids, and amino acids. These molecules are often utilized as the preferred carbon source by some microbes. For instance, beneficial rhizospheric bacteria prefer sugars and polymers like chitin, chitosan, glucan, glucosamine, etc., which they metabolize into organic acids like acetic acid, citric acid, formic acid, glycolic acid, lactic acid, malonic acid, oxalic acid, succinic acid, and more [228]. The substrate composition determines the fate of the microbial community composition. For example, chitin and chitosan, components of microbial exudates, modulate the Actinobacterial and Oxalobacteraceal community structure in the rhizosphere [229]. The amendment of chitin and chitosan increases the activity of chitinase enzymes, leading to the degradation into lower oligomers like N-acetylglucosamine, which is further catabolized into ammonia and other volatiles [230,231]. In this way, complex-sugar-based microbial exudates enhance the relative abundance of plant-growth-promoting rhizobacteria (e.g., *Bacillus* sp., *Pseudomonas* sp., and *Streptomyces* sp.) and other rhizospheric microbiota involved in the N-cycle, sugar degradation, and organic acid production.

Organic acids are another significant component of microbial exudates involved in restructuring the microbial community. They are associated with the mineralization process, and specific groups of microbes are engaged in different stages of the mineralization cycle. The exogenous application of organic acids supports the colonization of specific microbial groups and decreases species richness by lowering diversity indices, resulting in continuous changes in the microbiological community structure. For example, lactic acid favors the proliferation of members of the family Bacillaceae and Micrococcaceae, supporting biodegradation and enhancing soil fertility, leading to rapid changes in the microbiota composition. However, once the lactic acid is degraded, the previous microbial species return to the niche, while specific members of certain distinctive families like Pseudomonadaceae and Rhizobiaceae remain constantly present. Similar phenomena are also evident in citric acid, which favors the growth of members of the Clostridiaceae family, along with the presence of Pseudomonadaceae family members. Likewise, oxalic acid induces long-term changes in the bacterial community composition, with the dominance of members of Burkholderiaceae accompanied by plant-growth-promoting rhizobacteria [232]. In this way, the microbial community changes with a change in the chemical composition of microbial exudates.

Peptides and amino acids present in microbial exudates can also serve as nutritional sources of both carbon and nitrogen for the plant-associated microbiome. These molecules are generated through the proteolysis of complex proteinaceous compounds in the environment or as cellular efflux from microbial cells. Phylospheric and rhizospheric microbes can use these amino acids as organic nitrogen for their growth and proliferation. For instance, the exogenous application of L-α-amino-acid-based biostimulants containing biologically active free amino acids enhances the population of Actinobacteria in the rhizospheric soil of lettuce (*Lactuca sativa* L.) and also promotes the population of entomopathogenic fungi (*Beauveria* sp. and *Metarhizium* sp.) to suppress fungi and nematode infestation [233]. Similarly, the application of glutamic acid also modulates the core microbiome in the strawberry and tomato rhizosphere through an increase in the population of Streptomyces sp. [234]. Besides amino acids, protein hydrolysates can also reshape the plant microbiome [235], as their foliar application in lettuce promotes the colonization of epiphytic bacteria like *Enterobacter* sp., *Pantoea* sp., etc. [236]. In this way, peptides and amino acids can alter the microbial population structure in the rhizosphere and phyllosphere.

### 6.2. Microbial Exudates as Signaling Molecules for Other Microbes

Besides sugars and organic acids, the cell-free microbial exudates contain many small chemical compounds that act as intra- and inter-kingdom signaling molecules in microbial populations. They also influence interactions between plants and micro-organisms in the phyllosphere and rhizosphere. These compounds represent chemically and functionally diverse molecules, such as acyl homoserine lactones, amino acids, polymers, hormones, antimicrobials, etc.

Among them, N-acyl-homoserine lactones (N-AHLs) act as signal molecules that trigger quorum sensing (QS) mechanisms in plant-associated microbes, including beneficial and pathogenic species. QS is crucial for cellular communication within microbial groups or between groups, necessary for biofilm formation and gene activation for microbial function. The exogenous application of N-AHLs can change the alpha and beta diversity of the rhizospheric microbial population, as evident in *Panax ginseng*, with the dominance of beneficial species of Pseudomonas [237], thus playing a key role in shaping the soil microbiome.

Likewise, hormones are another crucial chemical compound found in cell-free microbial exudates, produced abundantly in the cultural filtrates of many plant-associated microbes. They act as ‘messengers’ or signaling compounds involved in many biological processes of plants, as well as microbes present in the phyllosphere and rhizosphere. Apart from supporting cellular and physiological processes in plants, they act as signaling compounds [238] and a nutrient source, influencing microbial community composition directly. For example, microbial IAA serves as a signaling molecule for biofilm formation through lipopolysaccharide (LPS) and extracellular polysaccharide (EPS) production in bacteria [239]. It also serves as a protectant against unfavorable conditions like heat, cold, osmotic, and oxidative stress [240], participates in microbial gene regulation in beneficial rhizobacteria like *Rhizobium etli* [241] and *Azospirillum brasilense* [242], induces antibiotic production in *Streptomyces* sp. [243], and acts as a substrate for microbial growth promotion [244] and chemotaxis in the population [245]. In this way, microbial hormones can also contribute to microbial diversity in the phyllosphere and rhizosphere.

### 6.3. Microbial Exudates Promote Niche Adaptation

The microbial ecology of the plant rhizosphere is very complex and is presented in terms of the diversity and composition of microbial taxa within it. Within a plant rhizospheric ecology, different microbial groups form small niches for their specific properties and metabolic activities [246]. The exudates of microbes, as well as plant origin, can greatly influence niche formation. Within a niche, strong competition, coexistence, and co-dependence among microbial species can lead to changes in the microbial community composition. Interestingly, the structure of niches can be determined through niche differentiation, niche competition, and the creation of new niches [247]. In niche differentiation, different microbial species can coexist in a niche due to their differential substrate preferences. In contrast, niche competition occurs when different microbial species have the same substrate preference, leading to the competitive exclusion of some species. Sometimes, a new niche is created only when end products or metabolites of one strain are released into the rhizosphere and used by another cross-feeding species, creating a new metabolic niche. The same phenomenon is frequently evident in the plant rhizosphere and phyllosphere when cell-free microbial exudates are applied exogenously. The nutritional enrichment of *Flaveria robusta* with sucrose + amino acids and sucrose alone enhances microbial diversity in its phyllosphere zone and niche partitioning between two bacteria, namely, *Pantoea* sp. and *Pseudomonas* sp., through cross-feeding interactions [248]. This co-dependent evolution of microbes shapes the plant microbiome. On the other hand, competition for nutrients and ecological niches is another phenomenon that drives microbial diversity. The exogenous application of microbial exudates also promotes microbial competition. Interestingly, some chemicals favor desirable soil microbial species to consume them as growth substrates and also deter other species, thus modulating the core microbiome of the rhizosphere. Further, siderophores and organic acids present in microbial exudates are another class of molecules that strengthen microbial competition and promote the selection of specific microbial species through the chelation of cations like Fe^3+^, Ca^2+^, Na^2+^, K^2+^, etc. They also help in the release of phosphates from insoluble phosphate compounds to make them available for the plant and associated microbes [249], thus changing their lifestyle (co-dependence). These molecules can also influence the evolution of microbial species, their selection by plants, and adaptation for root colonization. These ecological and evolutionary processes can strongly shape rhizospheric microbiota.

## 7. Limitations and Constraints

The utilization of microbial biostimulants in crop production is comparatively a new approach and is still lagging due to a lack of awareness regarding their potential. The strategy has gained prominence only in the past decades. The diverse nature and chemical composition of microbial active compounds have widened their utilization in various fields. However, several constraints need to be addressed before we commercialize any of the microbial exudates as biostimulants or bio-agents. The genetic basis of interaction between these biostimulants and plants is not fully explored, and the actual mechanism and mode of action of these biostimulants remain unclear. The diversity in microbial strains and their bioactive compounds makes it difficult to compare the effect of their application and evaluate the results. The relation between microbial biostimulants and how they alter or modify plant physiology is complex, and the lack of research illustrating the role and relevance of these molecules involved in the biostimulation process or their molecular mechanism involved in the bio-stimulatory action is a drawback for further research. The application of these metabolites has shown positive results in plant growth and development, protection against phytopathogenic microbes and abiotic stress, or mitigation of metal toxicity, especially in the invitro condition. However, replicating the same results at the field level is a bottleneck due to difficulties in producing suitable formulations for modern agriculture and also due to the failure of some exudates to be effective at a large scale. For instance, the field application of 3-pentanol and 2-butanol (VOCs) showed limited control over *P. syringae* [250]. Another constraint in the utilization of microbial biostimulants is that their effectiveness can be influenced by several factors, such as microbial strain, agricultural soil types, plant species, and concentration of the compounds. Their antagonistic effects cannot be neglected. Even though the European Regulation (EU) 2019/1009 includes micro-organisms under the fertilizer legislation, the rules and regulations regarding the categorization of biostimulants are vague, making the registration of any microbial product as a biostimulant difficult and complicated. Although microbial exudates hold a promising role in crop production, these constraints affect the development of microbial exudates as a potential biostimulant.

## 8. Conclusions and Prospects

The interaction between plant systems and microbes dates back to ancient times, resulting in an intricate relationship that can be beneficial, detrimental, or neutral. While numerous microbes are phytopathogenic, a good share of beneficial microbes interacts with the plant, positively influencing crop development. Microbes produce a diverse array of bioactive compounds/metabolites, allowing them to form an intricate relationship with the plant system. Various metabolites and exudates are released in the process, which can alter or modify plant physiology to improve its growth, development, and resilience against biotic and abiotic stress. Exudates secreted or released by microbes, such as phytohormones, exopolysaccharides, and VOCs, are mainly secondary metabolites of diverse chemical nature and composition, which play diverse roles in crop improvement and can be effective biostimulants. The need to minimize the use of chemical fertilizers and pesticides is imperative if sustainability, soil fertility conservation, and ecological balance are to be restored. The potential role of microbes and their exudates can prove to be essential components as bio-agents, bioremediators, or biostimulants in regenerative agriculture or organic farming systems.

Despite realizing the potential of microbes to some extent, there is a fundamental lack of investment in and implementation of their role in the agriculture sector. Thus, optimizing cultivation conditions, biochemically characterizing microbial biostimulants, conducting extensive research on their mode of action and mechanism, and determining the dose and concentration of the compounds and their effective formulation are necessary for developing microbial exudates as biostimulants at the commercial level. It is essential to qualitatively and quantitatively identify compounds secreted by microbes and explore their transcriptomics, metabolomics, and proteomics to characterize their biosynthetic pathways and maximize their utilization as potential biostimulants. Addressing these challenges and constraints will pave the way for realizing the full potential of microbial exudates as an essential component of sustainable and eco-friendly agriculture systems.

## Figures and Tables

**Table 2:** Microbial exudates in plant growth promotion and amelioration of biotic stress.

Microbes	Microbial Exudates	Mode of Action	References
*Azotobacter chroococcum*	Exopolysaccharide	Plant growth promotion in Faba bean	[130]
*Bacillus gibsonii* (PM11), *B. xiamenensis* (PM14)	Exopolysaccharide	Enhanced nutrient availability and plant growth of *Linum usitatissimum* by minimizing metal-induced stressed conditions	[131]
*Acinetobacter calcoaceticus* (9EU- LRNA-72), *Penicillium* sp. (EU-FTF-6)	Metabolites containing glycine betaine, proline, sugars, etc.	Increase in chlorophyll synthesis and decrease in lipid peroxidation	[132]
*Trichoderma harzianum* (M 10)	Harzianic acid (siderophore)	Induce expression of resistant genes (CC-NBS-LRR) in tomato	[133]
*Bacillus amyloliquefaciens* (FZB42)	Bacillomycin D (lipopeptide)	Degradation of mycotoxin production and disintegration of plasma membrane of *Fusarium graminearum* (head blight pathogen of wheat) through the production of reactive oxygen species (ROS)	[134]
*Aureobasidium pullulans*	VOCs (ethanol, 2-methylpropan-1-ol, 3-methylbutan-1-ol, and 2-phenylethanol)	Increases intracellular reactive oxygen species (ROS) accumulation, lipid peroxidation, and content leakage, thereby inhibiting *Botrytis cinerea* growth	[135]
*A. pullulans*	VOCs	Triggers lipid peroxidation and electrolyte leakage in *B. cinerea* and *Alternaria alternate*	[135]
*B. subtilis* (BS2)	Metabolites	Defense enzyme production such as peroxidase (PO), polyphenol oxidase (PPO), chitinase, and phenylalanine	[136]
*Pseudomonas furukawaii*, *P. plecoglossicida*, *P. alcaligenes*, *P. oleovarans*, *Leclercia adecarboxylata*, *Citrobacter youngae*, *Enterobacter cloacae*	Hydroxymate and catecholate	Antagonistic activities against different phytopathogens like *Rhizoctonia solani*, *Phythium* sp., *Fusarium oxysporum* in *Phaseolus vulgaris*, *Helianthus* sp., *Triticum astivum*, *Oryza sativa*	[137]
Microalgae	Polysaccharides	Phytostimulant property in tomato	[138]
*Arthospira platensis*	Polyamines	Regulation of gene expression and protein synthesis for the modulation of signal transduction	[139]
*Pseudomonas putida* (CRN-09), *Bacillus subtilis* (CRN-16)	Metabolites	Production of PO, PPO, beta 1,3-glucanse, chitinase, and phenylalanine ammonia lyase (PAL) against *Macrophomina phaseolina*	[140]
*B. subtilis*	VOCs (2,3-butanedione; 3-methylbutyric acid)	Antifungal activity (inhibited hyphal growth) against *Mucor circinelloides; Fusarium arcuatisporum; A. iridiaustralis*; *Colletotrichum fioriniae;* and reduced decay of wolfberry fruits	[141]
*Pseudomonas fluorescens* (G20-18)	Bacterial cytokinin	Activates plant resistance against pathogenic *P. syringae*	[142,143]
*P. fluorescens*(C7R12)	Pyoverdine siderophore	Enhanced root and shoot ratio in *Pisum sativum* by promoting plant iron nutrition	[144]
*Bacillus licheniformis* (DS3)	Hydroxymate	Biological agents against several fungal pathogens like *Aspergillus niger*, *Alternaria solani*, *Fusarium solani*, and *Fusarium oxysporium* in *Vigna mungo*	[145]
*Ascophyllum nodosum*	Complex polysaccharides (fucans and alginates)	The combination treatment of chitosan and *A. nodosum* liquid sea weed extract (containing complex polysaccharide) reduced the level of mycotoxins deoxynivalenol and sambucinol produced by *Fusarium graminearum* in wheat grains by inducing defense genes and enzymes.	[146]

**Table 3:** Microbial exudates mediated mitigation of abiotic stress.

Microbes	Microbial Exudates	Mode of Action	References
*Pseudomonas anguilliseptica* (SAW24)	Exopolysaccharide	Enhances biofilm stability under salinity stress and, thus, protecting the plant root system	[147]
*Azotobacter* sp. (AztRMD2)	Exopolysaccharide	Augment soil aggregate stability in rice under drought stress condition	[148]
*Bacillus endophyticus* (J13), *B. tequilensis* (J12)	Exopolysaccharide, IAA, cytokinin	Alleviation of osmotic stress in Arabidopsis	[149]
*Bacillus gibsonii* (PM11), *B. xiamenensis* (PM14)	Exopolysaccharide	Enhanced nutrient availability and plant growth of *Linum usitatissimum* by minimizing metal stress	[131]
*Leclercia adecarboxylata* (MO1)	Metabolites	Salinity stress tolerance in soybean via auxin biosynthesis	[150]
*Dermacoccus barathri* (MT2.1T), *D. profundi* (MT2.2T), and *D. nishinomiyaensis* (DSM20448T)	Hydroxamate and catechol-type siderophores	Increased tomato seedling and plant growth under saline condition	[151]
*Streptomyces acidiscabies* (E13)	Desferridoxine E Desferridoxine B Coelichelin	Nickel stress tolerance in cowpea through nickle sequesteration	[152]
*B. subtilis*	Endophytic siderophore	Enhanced growth and survivability of wheat under drought condition	[153]
*Pseudomonas citronellolis* strain *(SLP6)*	Hydroxymate siderophore	Significantly enhanced chlorophyll content, antioxidant enzyme production, and plant growth in *Helianthus annus* under salinity stress condition	[154]
*Halomonas* sp.	Exo1exopolysaccharide	In presence of arsenic, Exo1 EPSs favor metal ion sequestration by biosorption due to the negative charge matrix of the EPS and alleviated heavy metal stress in rice	[155]
*Pseudomonas pseudoalcaligenes*	VOCs (dimethyl disulfide, 2,3-butanediol, and 2-pentylfuran)	Drought tolerance in maize plants by reducing electrolyte leakage and malondialdehyde content, and increasing proline and phytohormone content	[156]
*Halobacillus* sp.(ADN1), *Halomonas* sp.(MAN5),and *Halobacillus* sp. (MAN6)	Exopolysaccharide	Retention of indole acetic acid and phosphate solubilization capacity under salinity and heavy metal stress (Cd, Ni, Hg, and Ag) to enhance root growth in *Sesuvium portulacastrum*	[157]
*Arthrobacter globiformis*(MSRC52), *Bacillus licheniformis*(MSRC76), *B. megaterium* (MSRC23)	Siderophore and IAA	Tolerance to salinity and high-temperature stress in olive trees	[158]
*B. velezensis* D3	ACC-deaminase, EPS and siderophore	Improved the growth and physiology of maize under drought stress throughout	[159]
*B. Cereus*	ACC-deaminase and EPS	Mitigation of heat stress in *Solanum lycopersicum* and improvement of physiological and biochemical traits	[160]

**Table 4:** Microbial exudate in degradation of xenobiotic compounds.

Xenobiotic	Microbe	Enzyme	Mechanism of Degradation	References
Atrazine	*Bjerkandera adusta*	Laccases, tyrosimases, manganese peroxidases (MnPs), manganese- independent peroxidases (MiPs), and lignin peroxidases	De-alkylation of atrazine resulting in removal efficiency of upto 92%.	[217]
Chlorpyrifos	*Cladosporium cladosporioides*	Chlorpyrifos hydrolase, pectin methylesterase (PME), and polygalacturonase (PG)	Responsible for pectin degradation by catalyzing the demethoxylation of the homogalacturonan chain of pectin to release methanol and acidic pectin	[218]
Atrazine Monocrotophos, DDT	*Fusarium* spp.	N-acetyltransferae and N-malonyltransferase	Detoxification and degradation of aromatic amines	[219]
Aromatic compounds, aliphatic hydrocarbons, PAHs	*Trichoderma harzianum*, *Aspergillus fumigatus*, *Cunninghamella elegans*, *Aspergillus niger*, *Penicillium* sp., *Cunninghamella elegans*, *Aspergillus ochraceus*, *Trametes versicolor*, *Penicillium* sp. RMA1 and RMA2, and Aspergillus sp. RFC-1	Lactase, lignin peroxidases (LiPs), MnPs, epoxide hydrolases cytochrome P450 monoxygenase, dioxygenases, protease, and lipase	By peripheral degradation pathways, organic pollutants are gradually transformed, and many intermediate products are formed	[220]
Lignin, polychlorinated biphenyls (PCBs), petroleum hydrocarbons, PAHs, trinitroluenes, industrial dye effluents, herbicides, and pesticides	*Trametes versicolor*, *Phanerochaete chrysosporium*, *Rigidoporous lignosus*, and *Pleurotus ostreatus*	Lignin peroxidase, versatile peroxidase, laccase, and manganese peroxidise	Formation of semi-quinone intermediate during the oxidation of lignin-derived hyroquinone by laccase. It cleaves C-C bonds and oxidizes benzyl alcohols to aldehydes or ketones.	[221,222]
Organophosphorus pesticide- Profenfos and Quinalphos	*Kosakonia oryzae strain* VITPSCQ3	Organophosphorous hydrolase and phosphatase	Hydrolytic cleavage of P–S bond in phosphorodithioate and phosphorothioate and P–O bond in phosphate-containing pesticides	[223]
Fipronil (Phenyl-pyrazole insecticide)	*Aspergillus glaucus*, *Bacillus frmus*, *B. thuringiensis*, *Bacillus* sp., *Paracoccus* sp., *Streptomyces rochei*,and *Stenotrophomonas acidaminiphila*	Ligninolytic enzyme MnPs, the cytochrome P450 enzyme, and esterase	Oxidation, reduction, and hydrolysis	[224]

## Data Availability

Not applicable.

## References

[1] Abdel Latef A.A.H., Abu Alhmad M.F., Kordrostami M., Abo-Baker A.-B.A.-E., Zakir A. (2020). Inoculation with *Azospirillumlipoferum* or *Azotobacterchroococcum* reinforces maize growth byimproving physiological activities under saline conditions. J. Plant Growth Regul..

[2] Mayak S., Tirosh T., Glick B.R. (2004). Plant growth promoting bacteria that confer resistance towater stress in tomato and pepper. Plant Sci..

[3] Hui L.J., Kim S.D. (2013). Induction of drought stress resistance by multi-functional PGPR *Bacillus licheniformis* K11 in pepper. Plant Pathol. J..

[4] Zhang H., Kim M.S., Sun Y., Dowd S.E., Shi H., Paré P.W. (2008). Soil bacteria confer plant salttolerance by tissue-specific regulation of the sodium transporter HKT1. Mol. Plant Microbe Interact..

[5] Atouei M.T., Pourbabaee A.A., Shorafa M. (2019). Alleviation of salinity stress on some growthparameters of wheat by exopolysaccharide-producing bacteria. Iran. J. Sci. Technol. Trans. Sci..

[6] Alami Y., Achouak W., Marol C., Heulin T. (2000). Rhizosphere soil aggregation and plant growthpromotion of sunflowers by an exopolysaccharide-producing Rhizobium sp. strain isolated from sunflower roots. Appl. Environ. Microbiol..

[7] Lu X., Liu S.F., Yue L., Zhao X., Zhang Y.B., Xie Z.K., Wang R.Y. (2018). Epsc involved in the encoding of exopolysaccharides produced by *Bacillus amyloliquefaciens* FZB42 act to boost the drought tolerance of *Arabidopsis thaliana*. Int. J. Mol. Sci..

[8] Cohen A.C., Travaglia C.N., Bottini R., Piccoli P.N. (2009). Participation of abscisic acid andgibberellins produced by endophytic Azospirillum in the alleviation of drought effects in maize. Botanique.

[9] Liu W., Mu W., Zhu B.Y., Du Y.C., Liu F. (2008). Antagonistic activities of volatiles from four strains of *Bacillus* spp. and *Paeni bacillus* spp. against soil-borne plant pathogens. Agric. Sci. China.

[10] Kang S.M., Radhakrishnan R., Khan A.L., Kim M.J., Park J.M., Kim B.R., Shin D.H., Lee I.J. (2014). Gibberellin secreting rhizobacterium, *Pseudomonas putida* H-2-3 modulates the hormonal andstress physiology of soybean to improve the plant growth under saline and drought conditions. Plant Physiol. Biochem..

[11] Thakur M., Medintz I.L., Walper S.A. (2019). Enzymatic bioremediation of organophosphatecompounds—Progress and remaining challenges. Front. Bioeng. Biotechnol..

[12] European Commission (2019). The European Parliament and the Council of the European Union Regulation (EU) 2019/1009 of the European Parliament and of the Council of 5 June 2019 laying down rules on the making available on the market of EU fertilising products and amending Regulation (EC) No. 1069/2009 and (EC) No. 1107/2009 and repealing Regulation. Off. J. Eur. Union.

[13] Castiglione A.M., Mannino G., Contartese V., Bertea C.M., Ertani A. (2021). Microbial biostimulants as response to modern agriculture needs: Composition, role and application of these innovative products. Plants.

[14] Veliz-Vallejos D.F., van Noorden G.E., Yuan M., Mathesius U. (2014). A SinorhizobiumMeliloti-Specific N-Acyl Homoserine Lactone Quorum-Sensing Signal Increases Nodule Numbers in Medicago Truncatula Independent of Autoregulation. Front. Plant Sci..

[15] Banchio E., Xie X., Zhang H., Paré P.W. (2009). Soil Bacteria Elevate Essential Oil Accumulation and Emissions in Sweet Basil. J. Agric. Food Chem..

[16] Hanif M.K., Malik K.A., Hameed S., Saddique M.J., Fatima K., Naqqash T., Majeed A., Iqbal M.J., Imran A. (2020). Growth Stimulatory Effect of AHL Producing *Serratia* spp. from Potato on Homologous and Non-Homologous Host Plants. Microbiol. Res..

[17] Shrestha A., Grimm M., Ojiro I., Krumwiede J., Schikora A. (2020). Impact of Quorum Sensing Molecules on Plant Growth and Immune System. Front. Microbiol..

[18] Kumar M., Mishra S., Dixit V., Kumar M., Agarwal L., Chauhan P.S., Nautiyal C.S. (2016). Synergistic Effect of *Pseudomonas putida* and *Bacillus amyloliquefaciens* Ameliorates Drought Stress in Chickpea (*Cicer arietinum* L.). Plant SignalBehav..

[19] Sultana S., Alam S., Karim M.M. (2021). Screening of Siderophore-Producing Salt-Tolerant Rhizobacteria Suitable for Supporting Plant Growth in Saline Soils with Iron Limitation. J. Agri. Food Res..

[20] Hofmann M., Heine T., Malik L., Hofmann S., Joffroy K., Senges C.H.R., Bandow J.E., Tischler D. (2021). Screening for Microbial Metal-Chelating Siderophores for the Removal of Metal Ions from Solutions. Microorganisms.

[21] Ahmed E., Holmstrom S.J.M. (2014). Siderophores in Environmental Research: Roles and Applications. Microb. Biotechnol..

[22] Schalk I.J., Hannauer M., Braud A. (2011). Mini Review New Roles for Bacterial. Environ. Microbiol..

[23] Rajkumar M., Ae N., Prasad M.N.V., Freitas H. (2010). Potential of Siderophore-Producing Bacteria for Improving Heavy Metal Phytoextraction. Trends Biotechnol..

[24] Ito T. (1993). Enzymatic Determination of Itoic Acid, a Bacillus subtilis Siderophore, and 2, 3-Dihydroxybenzoic Acid. Appl. Environ. Microbiol..

[25] Shah S., Karkhanis V., Desai A. (1992). Isolation and Characterization of Siderophore, with Antimicrobial Activity, from *Azospirillumlipoferum*. M. Curr. Microbiol..

[26] Tindale A.E., Mehrotra M., Ottem D., Page W.J. (2000). Dual Regulation of Catecholate Siderophore Biosynthesis in *Azotobactervinelandii* by Iron and Oxidative Stress. Microbiology.

[27] Eng-Wilmot D.L., Van der Helm D. (1980). Molecular and Crystal Structure of the Linear Tricatechol Siderophore, Agrobactin. J. Am. Chem. Soc..

[28] Storey E.P., Boghozian R., Little J.L., Lowman D.W., Chakraborty R. (2006). Characterization of ‘Schizokinen’; a Dihydroxamate-Type Siderophore Produced by *Rhizobium leguminosarum* IARI 917. BioMetals.

[29] Kumari S., Kiran S., Kumari S., Kumar P., Singh A. (2022). Optimization of Siderophore Production by *Bacillus subtilis* DR2 and Its Effect on Growth of *Coriandrum Sativum*. Russ. Agricult. Sci..

[30] Igiehon N.O., Babalola O.O., Aremu B.R. (2019). Genomic Insights into Plant Growth Promoting Rhizobia Capable of Enhancing Soybean Germination under Drought Stress. BMC Microbiol..

[31] Ghazy N., El-Nahrawy S. (2021). Siderophore Production by *Bacillus subtilis* MF497446 and *Pseudomonas koreensis* MG209738 and Their Efficacy in Controlling Cephalosporium maydis in Maize Plant. Arch. Microbiol..

[32] Lynch D., O’Brien J., Welch T., Clarke P., Cuív P.O., Crosa J.H., O’Connell M. (2001). Genetic Organization of the Region Encoding Regulation, Biosynthesis, and Transport of Rhizobactin 1021, a Siderophore Produced by *Sinorhizobium meliloti*. J.Bacteriol..

[33] Drechsel H., Tschierske M., Thieken A., Jung G., Zähner H., Winkelmann G. (1995). The Carboxylate Type *Siderophore rhizoferrin* and Its Analogs Produced by Directed Fermentation. J. Indust. Microbiol..

[34] Campestre M.P., Castagno L.N., Estrella M.J., Ruiz O.A. (2016). Lotus japonicus plants of the Gifu B-129 ecotype subjected to alkaline stress improve their Fe^2+^ bio-availability through inoculation with *Pantoea eucalypti* M91. J. Plant Physiol..

[35] Mishra A., Jha B., Rosenberg E., DeLong E.F., Thompson F., Lory S., Stackebrandt E. (2013). MicrobialExopolysaccharides. InThe Prokaryotes: Applied Bacteriology and Biotechnology.

[36] Schulz-Bohm K., Martín-Sánchez L., Garbeva P. (2017). Microbial Volatiles: Small Molecules with an Important Role in Intra- and Inter-Kingdom Interactions. Front. Microbiol..

[37] Margaritis A., Pace G.W., Moo-Young M. (1985). Microbial Polysaccharides. Comprehensive Biotechnology.

[38] Nandal K., Sehrawat A.R., Yadav A.S., Vashishat R.K., Boora K.S. (2005). High Temperature-Induced Changes in Exopolysaccharides, Lipopolysaccharides, and Protein Profile of Heat-Resistant Mutants of *Rhizobium* sp. (Cajanus). Microbiol. Res..

[39] Upadhyay S.K., Singh J.S., Singh D.P. (2011). Exopolysaccharide-Producing Plant Growth-Promoting Rhizobacteria under Salinity Condition. Pedosphere.

[40] Pulsawat W., Leksawasdi N., Rogers P.L., Foster L.J.R. (2003). Anions Effects on Biosorption of Mn(II) by Extracellular Polymeric Substance (EPS) from *Rhizobium etli*. Biotechnol. Lett..

[41] Morcillo R.J.L., Manzanera M. (2021). The Effects of Plant-Associated Bacterial Exopolysaccharides on Plant Abiotic Stress Tolerance. Metabolites.

[42] Carminati A., Schneider C.L., Moradi A.B., Zarebanadkouki M., Vetterlein D., Vogel H.J., Hildebrandt A., Weller U., Schüler L., Oswald S.E. (2011). How the Rhizosphere May Favor Water Availability to Roots. Vadose Zone J..

[43] Naseem H., Bano A. (2014). Role of Plant Growth-Promoting Rhizobacteria and Their Exopolysaccharide in Drought Tolerance of Maize. J. Plant Interact..

[44] Dar A., Zahir Z.A., Iqbal M., Mehmood A., Javed A., Hussain A., Ahmad M. (2021). Efficacy of Rhizobacterial Exopolysaccharides in Improving Plant Growth, Physiology, and Soil Properties. Environ. Monit. Assess..

[45] Ishii S., Koki J., Unno H., Hori K. (2004). Two Morphological Types of Cell Appendages on a Strongly Adhesive Bacterium, *Acinetobacter* sp. Strain Tol 5. Appl. Environ. Microbiol..

[46] Fujishige N.A., Kapadia N.N., Hirsch A.M. (2006). A Feeling for the Micro-Organism: Structure on a Small Scale. Biofilms on Plant Roots. Bot. J. Linn. Soc..

[47] Hori K., Matsumoto S. (2010). Bacterial Adhesion: From Mechanism to Control. Biochem. Eng. J..

[48] Fazli M., Almblad H., Rybtke M.L., Givskov M., Eberl L., Tolker-Nielsen T. (2014). Regulation of Biofilm Formation in *Pseudomonas* and *Burkholderia* Species. Environ. Microbiol..

[49] Cheng C., Shang-Guan W., He L., Sheng X. (2020). Effect of Exopolysaccharide-Producing Bacteria on Water-Stable Macro-Aggregate Formation in Soil. Geomicrobiol. J..

[50] Zheng W., Zeng S., Bais H., LaManna J.M., Hussey D.S., Jacobson D.L., Jin Y. (2018). Plant Growth-Promoting Rhizobacteria (PGPR) Reduce Evaporation and Increase Soil Water Retention. Water Resour. Res..

[51] Benard P., Bickel S., Kaestner A., Lehmann P., Carminati A. (2023). Extracellular Polymeric Substances from Soil-Grown Bacteria Delay Evaporative Drying. Adv. Water Resour..

[52] Kudoyarova G., Arkhipova T., Korshunova T., Bakaeva M., Loginov O., Dodd I.C. (2019). Phytohormone Mediation of Interactions between Plants and Non-Symbiotic Growth-Promoting Bacteria under Edaphic Stresses. Front. Plant Sci..

[53] Kudoyarova G.R., Melentiev A.I., Martynenko E.V., Timergalina L.N., Arkhipova T.N., Shendel G.V., Kuz’mina L.Y., Dodd I.C., Veselov S.Y. (2014). Cytokinin Producing Bacteria Stimulate Amino Acid Deposition by Wheat Roots. Plant Physiol. Biochem..

[54] Wheatley R., Hackett C., Bruce A., Kundzewicz A. (1997). Effect of Substrate Composition on Production of Volatile Organic Compounds from *Trichoderma* spp. Inhibitory to Wood Decay Fungi. Int. Biodeterior. Biodegrad..

[55] Chiron N., Micherlot D. (2005). Odeurs des Champignons: Chimie et Rôle dans les Interactions Biotiques-une Revue. Cryptogam.-Mycol..

[56] Morath S.U., Hung R., Bennett J.W. (2012). Fungal Volatile Organic Compounds: A Review with Emphasis on Their Biotechnological Potential. Fungal Biol. Rev..

[57] Schulz S., Dickschat J.S. (2007). Bacterial Volatiles: The Smell of Small Organisms. Nat. Prod. Rep..

[58] Blom D., Fabbri C., Connor E.C., Schiestl F.P., Klauser D.R., Boller T., Eberl L., Weisskopf L. (2011). Production of Plant Growth Modulating Volatiles Is Widespread among Rhizosphere Bacteria and Strongly Depends on Culture Conditions. Environ. Microbiol..

[59] Peñuelas J., Asensio D., Tholl D., Wenke K., Rosenkranz M., Piechulla B., Schnitzler J. (2014). Biogenic Volatile Emissions from the Soil. Plant Cell Environ..

[60] Schenkel D., Lemfack M.C., Piechulla B., Splivallo R. (2015). A Metaanalysis Approach for Assessing the Diversity and Specificity of Belowground Root and Microbial Volatiles. Front. Plant Sci..

[61] Bennett J.W., Hung R., Lee S., Padhi S., Hock B. (2012). Fungal and Bacterial Volatile Organic Compounds: An Overview and Their Role as Ecological Signaling Agents. Fungal Associations.

[62] Ryu C.M., Farag M.A., Hu C.H., Reddy M.S., Wei H.X., Paré P.W., Kloepper J.W. (2003). Bacterial Volatiles Promote Growth in Arabidopsis. Proc. Natl. Acad. Sci. USA.

[63] Zhao X., Zhou J., Tian R., Liu Y. (2022). Microbial Volatile Organic Compounds: Antifungal Mechanisms, Applications, and Challenges. Front. Microbiol..

[64] Li H., Bölscher T., Winnick M., Tfaily M.M., Cardon Z.G., Keiluweit M. (2021). Simple Plant and Microbial Exudates Destabilize Mineral-Associated Organic Matter via Multiple Pathways. Environ. Sci. Technol..

[65] Chari N.R., Taylor B.N. (2022). Soil Organic Matter Formation and Loss Are Mediated by Root Exudates in a Temperate Forest. Nat. Geosci..

[66] Nwachukwu B.C., Ayangbenro A.S., Babalola O.O. (2021). Elucidating the Rhizosphere Associated Bacteria for Environmental Sustainability. Agriculture.

[67] Jilling A., Keiluweit M., Contosta A.R., Frey S., Schimel J., Schnecker J., Smith R.G., Tiemann L., Grandy A.S. (2018). Minerals in the Rhizosphere: Overlooked Mediators of Soil Nitrogen Availability to Plants and Microbes. Biogeochemistry.

[68] Walton C.L., Khalid M., Bible A.N., Kertesz V., Retterer S.T., Morrell-Falvey J., Cahill J.F. (2022). In Situ Detection of Amino Acids from Bacterial Biofilms and Plant Root Exudates by Liquid Microjunction Surface-Sampling Probe Mass Spectrometry. J. Am. Soc. Mass. Spectrom..

[69] Wang Y., Luo D., Xiong Z., Wang Z., Gao M. (2023). Changes in rhizosphere phosphorus fractions and phosphate-mineralizing microbial populations in acid soil as influenced by organic acid exudation. Soil. Tillage Res..

[70] Romera F.J., García M.J., Lucena C., Martínez-Medina A., Aparicio M.A., Ramos J., Alcántara E., Angulo M., Pérez-Vicente R. (2019). Induced systemic resistance (ISR) and Fe deficiency responses in dicot plants. Front. Plant Sci..

[71] Zhang J.L., Tang W.L., Huang Q.R., Li Y.Z., Wei M.L., Jiang L.L., Liu C., Yu X., Zhu H.W., Chen G.Z. (2021). Trichoderma: A treasure house of structurally diverse secondary metabolites with medicinal importance. Front. Microb..

[72] Ray S., Singh P., Singh J., Singh S., Sarma B.K., Singh H.B. (2023). Killed fungal pathogen triggers antifungal metabolites in *Alcaligenes faecalis* for plant defense. Physiol. Mol. Plant Pathol..

[73] Tamandegani P.R., Marik T., Zafari D., Balázs D., Vágvölgyi C., Szekeres A., Kredics L. (2020). Changes in Peptaibol Production of Trichoderma Species during In Vitro Antagonistic Interactions with Fungal Plant Pathogens. Biomolecules.

[74] Shamikh Y.I., El Shamy A.A., Gaber Y., Abdelmohsen U.R., Madkour H.A., Horn H., Hassan H.M., Elmaidomy A.H., Alkhalifah D.H.M., Hozzein W.N. (2020). Actinomycetes from the Red Sea sponge *Coscinodermamathewsi*: Isolation, diversity, and potential for bioactive compounds discovery. Microorganisms.

[75] Ugena L., Hýlová A., Podlešáková K., Humplík J.F., Doležal K., De Diego N., Spíchal L. (2018). Characterization of biostimulant mode of action using novel multi-trait high-throughput screening of *Arabidopsis* germination and rosette growth. Front. Plant Sci..

[76] Ogunsanya H.Y., Motti P., Li J., Trinh H.K., Xu L., Bernaert N., Van Droogenbroeck B., Murvanidze N., Werbrouck S.P., Mangelinckx S. (2022). Belgian endive-derived biostimulants promote shoot and root growth in vitro. Sci. Rep..

[77] Saporta R., Bou C., Frías V., Mulet J.M. (2019). A method for a fast evaluation of the biostimulant potential of different natural extracts for promoting growth or tolerance against abiotic stress. Agronomy.

[78] Jiménez-Arias D., Morales-Sierra S., Borges A.A., Herrera A.J., Luis J.C. (2022). New biostimulants screening method for crop seedlings under water deficit stress. Agronomy.

[79] Hines S., van der Zwan T., Shiell K., Shotton K., Prithiviraj B. (2021). Alkaline extract of the seaweed *Ascophyllum nodosum* stimulates arbuscular mycorrhizal fungi and their endomycorrhization of plant roots. Sci. Rep..

[80] Meher J., Lenka S., Sarkar A., Sarma B.K. (2023). Transcriptional regulation of *OsWRKY* genes in response to individual and overlapped challenges of *Magnaportheoryzae* and drought in indica genotypes of rice. Environ. Exp. Bot..

[81] Kumar S., Mohapatra T. (2021). Dynamics of DNA Methylation and Its Functions in Plant Growth and Development. Front. Plant Sci..

[82] Quievreux M., Falesse W., Lengrand S., Delaplace P., Dieryck B., Dumont de Chassart S., Dumont de Chassart T., Legrève A., Jardin P. Testing biostimulants for validating the claims: A multi-level analysis. Proceedings of the 5th Biostimulants World Congress.

[83] De Diego N., Spíchal L. (2022). Presence and future of plant phenotyping approaches in biostimulant research and development. J. Exp. Bot..

[84] Khiralla A., Spina R., Varbanov M., Philippot S., Lemiere P., Slezack-Deschaumes S., André P., Mohamed I., Yagi S.M., Laurain-Mattar D. (2020). Evaluation of antiviral, antibacterial and antiproliferative activities of the endophytic fungus *Curvulariapapendorfii*, and isolation of a new polyhydroxyacid. Microorganisms.

[85] Papaianni M., Ricciardelli A., Fulgione A., d’Errico G., Zoina A., Lorito M., Woo S.L., Vinale F., Capparelli R. (2020). Antibiofilm Activity of a Trichoderma Metabolite against *Xanthomonas campestris* pv. *campestris*, Alone and in Association with a Phage. Microorganisms.

[86] Voitsekhovskaia I., Paulus C., Dahlem C., Rebets Y., Nadmid S., Zapp J., Axenov-Gribanov D., Rückert C., Timofeyev M., Kalinowski J. (2020). Baikalomycins AC, New Aquayamycin-type angucyclines isolated from Lake Baikal derived *Streptomyces* sp. IB201691-2A. Microorganisms.

[87] Hamed A.A., Soldatou S., Qader M.M., Arjunan S., Miranda K.J., Casolari F., Pavesi C., Diyaolu O.A., Thissera B., Eshelli M. (2020). Screening fungal endophytes derived from under-explored Egyptian marine habitats for antimicrobial and antioxidant properties in factionalised textiles. Microorganisms.

[88] Hwang S., Le L.T.H.L., Jo S.I., Shin J., Lee M.J., Oh D.C. (2020). Pentaminomycins C–E: Cyclic pentapeptides as autophagy inducers from a mealworm beetle gut bacterium. Microorganisms.

[89] Liu L., Hu Z., Li S., Yang H., Li S., Lv C., Zaynab M., Cheng C.H., Chen H., Yang X. (2020). Comparative transcriptomic analysis uncovers genes responsible for the DHA enhancement in the mutant *Aurantiochytrium* sp.. Microorganisms.

[90] Wolff P.B. (2020). Genomics-Driven Discovery, Characterization, and Engineering of Fungal Secondary Metabolites. Ph.D. Thesis.

[91] Ding Z., Wang X., Kong F.D., Huang H.M., Zhao Y.N., Liu M., Wang Z.P., Han J. (2020). Overexpression of global regulator talae1 leads to the discovery of new antifungal polyketides from endophytic fungus *Trichoderma afroharzianum*. Front. Microbiol..

[92] Staropoli A., Iacomino G., De Cicco P., Woo S.L., Di Costanzo L., Vinale F. (2023). Induced secondary metabolites of the beneficial fungus *Trichoderma harzianum* M10 through OSMAC approach. Chem. Biol. Technol. Agric..

[93] Hifnawy S., Hassan M., Mohammed H.M., Fouda R.M., Sayed M., Hamed A.M.A., AbouZid A.F., Rateb S., Alhadrami M.E., Abdelmohsen U.R. (2020). Induction of antibacterial metabolites by co-cultivation of two red-sea-sponge-associated actinomycetes *Micromonospora* sp. UR56 and *Actinokinespora* sp. EG49. Mar. Drugs.

[94] Liang L., Wang G., Haltli B., Marchbank D.H., Stryhn H., Correa H., Kerr R.G. (2020). Metabolomic comparison and assessment of co-cultivation and a heat-killed inducer strategy in activation of cryptic biosynthetic pathways. J. Nat. Prod..

[95] Jomori T., Hara Y., Sasaoka M., Harada K., Setiawan A., Hirata K., Kimishima A., Arai M. (2020). *Mycobacterium smegmatis* alters the production of secondary metabolites by marine-derived *Aspergillus niger*. J. Nat. Med..

[96] Lombardi N., Salzano A.M., Troise A.D., Scaloni A., Vitaglione P., Vinale F., Marra R., Caira S., Lorito M., d’Errico G. (2020). Effect of Trichoderma bioactive metabolite treatments on the production, quality, and protein profile of strawberry fruits. J. Agric. Food Chem..

[97] Shi Z.Z., Liu X.H., Li X.N., Ji N.Y. (2020). Antifungal and antimicroalgal trichothecene sesquiterpenes from the marine algicolous fungus *Trichoderma brevicompactum* A-DL-9-2. J. Agric. Food Chem..

[98] Yamazaki H., Takahashi O., Kirikoshi R., Yagi A., Ogasawara T., Bunya Y., Rotinsulu H., Uchida R., Namikoshi M. (2020). Epipolythiodiketopiperazine and trichothecene derivatives from the NaI-containing fermentation of marine-derived *Trichoderma* cf. brevicompactum. J. Antibiot..

[99] Du F.Y., Ju G.L., Xiao L., Zhou Y.M., Wu X. (2020). Sesquiterpenes and cyclodepsipeptides from marine-derived fungus *Trichoderma longibrachiatum* and their antagonistic activities against soil-borne pathogens. Mar. Drugs.

[100] Li W.Y., Liu Y., Lin Y.T., Liu Y.C., Guo K., Li X.N., Luo S.H., Li S.H. (2020). Antibacterial harziane diterpenoids from a fungal symbiont *Trichoderma atroviride* isolated from *Colquhounia coccinea* var. mollis. Phytochemistry.

[101] Miyano R., Matsuo H., Mokudai T., Noguchi Y., Higo M., Nonaka K., Niwano Y., Sunazuka T., Shiomi K., Takahashi Y. (2020). Trichothioneic acid, a new antioxidant compound produced by the fungal strain *Trichoderma virens* FKI-7573. J. Biosci. Bioeng..

[102] Zhang S., Sun F., Liu L., Bao L., Fang W., Yin C., Zhang Y. (2020). Dragonfly associated *Trichoderma harzianum* QTYC77 is not only a potential biological control agent of *Fusarium oxysporum* f. sp. *cucumerinum* but also a source of new antibacterial agents. J. Agric. Food Chem..

[103] Zhao D.L., Zhang X.F., Huang R.H., Wang D., Wang X.Q., Li Y.Q., Zheng C.J., Zhang P., Zhang C.S. (2020). Antifungal nafuredin and epithiodiketopiperazine derivatives from the mangrove-derived fungus *Trichoderma harzianum* D13. Front. Microbiol..

[104] Duzan H.M., Mabood F., Zhou X., Souleimanov A., Smith D.L. (2005). Nod factor induces soybean resistance to powdery mildew. Plant Physiol. Biochem..

[105] Choi H.K., Song G.C., Yi H.S., Ryu C.M. (2014). Field evaluation of the bacterial volatile derivative 3-pentanol in priming for induced resistance in pepper. J. Chem. Ecol..

[106] Prudent M., Salon C., Smith D.L., Emery R.J.N. (2016). Nod factor supply under water stress conditions modulates cytokinin biosynthesis and enhances nodule formation and N nutrition in soybean. Plant Signal. Behav..

[107] Piechulla B., Lemfack M.C., Kai M. (2017). Effects of discrete bioactive microbial volatiles on plants and fungi. Plant Cell Environ..

[108] Zhou D., Huang X.F., Chaparro J.M., Badri D.V., Manter D.K., Vivanco J.M., Guo J. (2016). Root and bacterial secretions regulate the interaction between plants and PGPR leading to distinct plant growth promotion effects. Plant Soil..

[109] Zhao Q., Yang X.Y., Li Y., Liu F., Cao X.Y., Jia Z.H., Song S.S. (2020). N-3-oxo-hexanoyl-homoserine lactone, a bacterial quorum sensing signal, enhances salt tolerance in *Arabidopsis* and wheat. Bot. Stud..

[110] Shrestha A., Elhady A., Adss S., Wehner G., Böttcher C., Heuer H., Ordon F., Schikora A. (2019). Genetic differences in barley govern the responsiveness to N-Acyl homoserine lactone. Phytobiomes J..

[111] Sayyed R.Z., Reddy M.S. (2021). Bacterial Plant Biostimulants: A sustainable way toward improving growth, productivity and health of crops. Sustainability.

[112] Kumar D.H., Aloke P. (2020). Role of biostimulants in crop production: An overview. Int. J. Agric. Sci. Vet. Med..

[113] Mine A., Sato M., Tsuda K. (2014). Toward a systems understanding of plant–microbe interactions. Front. Plant Sci..

[114] Ruzzi M., Aroca R. (2015). Plant growth-promoting rhizobacteria act as biostimulants in horticulture. Sci. Hortic..

[115] Paradikovic N., Teklic T., Zeljkovic S., Lisjak M., Spoljarevic M. (2019). Biostimulants research in some horticultural plant species—A review. Food Energy Secur..

[116] Kour D., Rana K.L., Yadav A.N., Yadav N., Kumar M., Kumar V., Vyas P., Dhaliwal H.S., Saxena A.K. (2020). Microbial biofertilizers: Bioresources and eco-friendly technologies for agricultural and environmental sustainability. Biocatal. Agric. Biotechnol..

[117] Santos-Torres M., Romero-Perdomo F., Mendoza-Labrador J., Gutiérrez A.Y., Vargas C., Castro-Rincon E., Caro-Quintero A., Uribe-Velez D., Estrada-Bonilla G.A. (2021). Genomic and phenotypic analysis of rock phosphate-solubilizing rhizobacteria. Rhizosphere.

[118] Hii Y.S., Yen San C., Lau S.W., Danquah M.K. (2020). Isolation and characterisation of phosphate solubilizing microorganisms from peat. Biocatal. Agric. Biotechnol..

[119] Santos M.S., Nogueira M.A., Hungria M. (2021). Outstanding impact of *Azospirillumbrasilense* strains ab-v5 and ab-v6 on Brazilian agriculture: Lessons that farmers are receptive to adopt new microbial inoculants. Rev. Bras. Cienc. Solo..

[120] Radhakrishnan R., Hashem A., Abd Allah E.F. (2017). *Bacillus*: A biological tool for crop improvement through bio-molecular changes in adverse environments. Front. Physiol..

[121] Kang S.M., Khan A.L., Waqas M., You Y.H., Kim J.H., Kim J.G., Hamayun M., Lee I.J. (2014). Plant growth-promoting rhizobacteria reduce adverse effects of salinity and osmotic stress by regulating phytohormones and antioxidants in *Cucumis sativus*. J. Plant Interact..

[122] Okada K., Abe H., Arimura G.I. (2015). Jasmonates induce both defense responses and communication in monocotyledonous and dicotyledonous plants. Plant Cell Physiol..

[123] Rijavec T., Lapanje A. (2016). Hydrogen cyanide in the rhizosphere: Not suppressing plant pathogens, but rather regulating availability of phosphate. Front. Microbiol..

[124] Llorente B.E., Alasia M.A., Larraburu E.E. (2016). Biofertilization with *Azospirillumbrasilense* improves in vitro culture of *Handroanthusochraceus*, a forestry, ornamental and medicinal plant. New Biotechnol..

[125] Lim J.H., Kim S.D. (2009). Synergistic plant growth promotion by the indigenous auxins producing PGPR *Bacillus subtilis* AH18 and *Bacillus licheniformis* K11. J. Korean Soc. Appl. Biol. Chem..

[126] Ortiz-Castro R., Campos-Garcıa J., Lopez-Bucio J. (2020). *Pseudomonas putida* and *Pseudomonas fluorescens* influence *Arabidopsis* root system architecture through an auxin response mediated by bioactive cyclodipeptides. J. Plant Growth Regul..

[127] Yadav S., Kaushik R., Saxena A.K., Arora D.K. (2011). Diversity and phylogeny of plant growth-promoting bacilli from moderately acidic soil. J. Basic. Microbiol..

[128] Hellequin E., Monard C., Chorin M., Le Bris N., Daburon V., Klarzynski O., Binet F. (2020). Responses of active soil microorganisms facing a soil biostimulant input compared to plant legacy effects. Sci. Rep..

[129] Jha Y., Subramanian R.B. (2014). PGPR regulate the caspase-like activity, programmed cell death, and antioxidant enzyme activity in paddy under salinity. Physiol. Mol. Biol. Plants.

[130] El-Ghany M.F.A., Attia M. (2020). Effect of exopolysaccharide-producing bacteria and melatonin on faba bean production in saline and non-saline soil. Agronomy.

[131] Zainab N., Din B.U., Javed M.T., Afridi M.S., Mukhtar T., Kamran M.A., Khan A.A., Ali J., Jatoi W.N., Munis M.F.H. (2020). Deciphering metal toxicity responses of flax (*Linumusitatissimum* L.) with exopolysaccharide and ACC-deaminase producing bacteria in industrially contaminated soils. Plant Physiol. Biochem..

[132] Kour D., Rana K.L., Yadav A.N., Sheikh I., Kumar V., Dhaliwal H.S., Saxena A.K. (2020). Amelioration of drought stress in Foxtail millet (*Setariaitalica* L.) by P-solubilizing drought-tolerant microbes with multifarious plant growth promoting attributes. Environ. Sustain..

[133] Manganiello G., Sacco A., Ercolano M.R., Vinale F., Lanzuise S., Pascale A., Napolitano M., Lombardi N., Lorito M., Woo S.L. (2018). Modulation of tomato response to *Rhizoctonia solani* by *Trichoderma harzianum* and its secondary metabolite harzianic acid. Front. Microbiol..

[134] Gu Q., Yang Y., Yuan Q., Shi G., Wu L., Lou Z., Huo R., Wu H., Borriss R., Gao X. (2017). Bacillomycin D produced by *Bacillus amyloliquefaciens* is involved in the antagonistic interaction with the plant-pathogenic fungus *Fusarium graminearum*. Appl. Environ. Microbiol..

[135] Don S.M.Y., Schmidtke L.M., Gambetta J.M., Steel C.C.J.R.I.M. (2020). Volatile organic compounds produced by *Aureobasidium pullulans* induce electrolyte loss and oxidative stress in *Botrytis cinerea* and *Alternaria alternata*. Res. Microbiol..

[136] Meena M., Zehra A. (2019). Tomato: A model plant to study plant-pathogen interactions. Food Sci. Nutr. Technol..

[137] Khaing A., Theint W.T., Thi O.K., Fu P. (2021). Antagonistic activity of indigenous rhizobacteria through biosynthesis of indole-3-acetic acid (IAA), hydrogen cyanide (HCN), and siderophores. Aust. J. Biotechnol. Bioeng..

[138] Rachidi F., Benhima R., Sbabou L., El Arroussi H. (2020). Microalgae polysaccharides bio-stimulating effect on tomato plants: Growth and metabolic distribution. Biotechnol. Rep..

[139] Jantaro S., Kanwal S., Rastogi R.P., Madamwar D. (2017). Low-Molecular-Weight Nitrogenous Compounds (GABA and Polyamines) in Blue-Green Algae. Algal Green Chemistry.

[140] Sharma C.K., Vishnoi V.K., Dubey R.C., Maheshwari D.K. (2018). A twin rhizospheric bacterial consortium induces systemic resistance to a phytopathogen *Macrophominaphaseolina* in mung bean. Rhizosphere.

[141] Ling L., Zhao Y., Tu Y., Yang C., Ma W., Feng S., Lu L., Zhang J. (2021). The inhibitory effect of volatile organic compounds produced by *Bacillus subtilis* CL2 on pathogenic fungi of wolfberry. J. Basic. Microbiol..

[142] Großkinsky D.K., Tafner R., Moreno M.V., Stenglein S.A., García de Salamone I.E., Nelson L.M., Novák O., Strnad M., Van der Graaff E., Roitsch T. (2016). Cytokinin production by *Pseudomonas fluorescens* G20–18 determines biocontrol activity against *Pseudomonas syringae* in *Arabidopsis*. Sci. Rep..

[143] Akhtar S.S., Mekureyaw M.F., Pandey C., Roitsch T. (2020). Role of cytokinins for interactions of plants with microbial pathogens and pest insects. Front. Plant Sci..

[144] Lurthy T., Cantat C., Jeudy C., Declerck P., Gallardo K., Barraud C., Leroy F., Ourry A., Lemanceau P., Salon C. (2020). Impact of bacterial siderophores on iron status and ionome in pea. Front. Plant Sci..

[145] Silpa D., Brahmaji R.P., Kranthi K.G. (2018). Biocontrol activity of Siderophores producing *Bacillus licheniformis* DS3 against several pathogenic fungi in Black gram [*Vigna mungo* (L.) *hepper*]. Int. J. Cur. Res..

[146] Gunupuru L.R., Patel J.S., Sumarah M.W., Renaud J.B., Mantin E.G., Prithiviraj B. (2019). A plant biostimulant made from the marine brown algae *Ascophyllum nodosum* and chitosan reduce *Fusarium* head blight and mycotoxin contamination in wheat. PLoS ONE.

[147] Mohammed A.F. (2018). Effectiveness of exopolysaccharides and biofilm forming plant growth promoting rhizobacteria on salinity tolerance of faba bean (*Viciafaba* L.). Afr. J. Microbiol. Res..

[148] Sivapriya S.L., Priya P.R. (2017). Selection of Hyper Exopolysaccharide Producing and Cyst Forming Azotobacter Isolates for Better Survival under Stress Conditions. Int. J. Curr. Microbiol. App. Sci..

[149] Ghosh D., Gupta A., Mohapatra S. (2019). A comparative analysis of exopolysaccharide and phytohormone secretions by four drought-tolerant rhizobacterial strains and their impact on osmotic-stress mitigation in *Arabidopsis thaliana*. World J. Microbiol. Biotechnol..

[150] Khan M.A., Asaf S., Khan A.L., Ullah I., Ali S., Kang S.M., Lee I.J. (2019). Alleviation of salt stress response in soybean plants with the endophytic bacterial isolate *Curtobacterium* sp. SAK1. Ann. Microbiol..

[151] Rangseekaew P., Barros-Rodriguez A., Pathom-aree W., Manzanera M. (2021). Deep-Sea actinobacteria mitigate salinity stress in tomato seedlings and their biosafety testing. Plants.

[152] Sathya A., Vijayabharathi R., Gopalakrishnan S. (2017). Plant growth-promoting actinobacteria: A new strategy for enhancing sustainable production and protection of grain legumes. 3Biotech.

[153] Silaochkina O., Garshina D., Pusenkova L. Effect of endophytic *Bacillus subtilis* on drought stress tolerance of *Triticum aestivum* plants of steppe volga and forest-steppe west siberian agroecological groups. Proceedings of the 2nd International Conference Plants and Microbes: The Future of Biotechnology.

[154] Silambarasan S., Logeswari P., Valentine A., Cornejo P., Kannan V.R. (2020). *Pseudomonas citronellolis* strain SLP6 enhances the phytoremediation efficiency of *Helianthus annuus* in copper contaminated soils under salinity stress. Plant Soil..

[155] Mukherjee P., Mitra A., Roy M. (2019). *Halomonas* rhizobacteria of *Avicennia marina* of Indian Sundarbans promote rice growth under saline and heavy metal stresses through exopolysaccharide production. Front. Microbiol..

[156] Yasmin H., Rashid U., Hassan M.N., Nosheen A., Naz R., Ilyas N., Sajjad M., Azmat A., Alyemeni M.N. (2021). Volatile organic compounds produced by *Pseudomonas pseudoalcaligenes* alleviated drought stress by modulating defense system in maize (*Zea mays* L.). Physiol. Planta.

[157] Desale P., Patel B., Singh S., Malhotra A., Nawani N. (2014). Plant growth promoting properties of *Halobacillus* sp. and *Halomonas* sp. in presence of salinity and heavy metals. J. Basic. Microbiol..

[158] Lahsini A.I., Sallami A., Obtel M., Douira A., El Modafar C., Benkerroum N., Talbi C., Chakhchar A., Filali-Maltouf A. (2022). Isolation and molecular identification of an indigenous abiotic stress-tolerant plant growth-promoting rhizobacteria from the rhizosphere of the olive tree in southern Morocco. Rhizosphere..

[159] Nadeem S.M., Ahmad M., Tufail M.A., Asghar H.N., Nazli F., Zahir Z.A. (2021). Appraising the potential of EPS-producing rhizobacteria with ACC-deaminase activity to improve growth and physiology of maize under drought stress. Physiol. Plant.

[160] Mukhtar T., Rehman S.U., Smith D., Sultan T., Seleiman M.F., Alsadon A.A., Amna A.S., Chaudhary H.J., Solieman T.H., Ibrahim A.A. (2020). Mitigation of heat stress in *Solanum lycopersicum* L. by ACC-deaminase and exopolysaccharide producing *Bacillus cereus*: Effects on biochemical profiling. Sustainability.

[161] Chattopadhyay A., Purohit J., Tiwari K.K., Deshmukh R. (2019). Targeting transcription factors for plant disease resistance: Shifting paradigm. Curr. Sci..

[162] Yuan J., Raza W., Shen Q., Huang Q. (2012). Antifungal activity of *Bacillus amyloliquefaciens* NJN-6 volatile compounds against *Fusarium oxysporum* f. sp. *cubense*. Appl. Environ. Microbiol..

[163] Cordero P., Principe A., Jofre E., Mori G., Fischer S. (2014). Inhibition of the phytopathogenic fungus *Fusarium proliferatum* by volatile compounds produced by *Pseudomonas*. Arch. Microbiol..

[164] Teli B., Purohit J., Rashid M.M., Jailani A.A.K., Chattopadhyay A. (2020). Omics insight on *Fusarium* head blight of wheat for translational research perspective. Curr. Genom..

[165] Giorgio A., De Stradis A., Lo Cantore P., Iacobellis N.S. (2015). Biocide effects of volatile organic compounds produced by potential biocontrol rhizobacteria on *Sclerotinia sclerotiorum*. Front. Microbiol..

[166] Groenhagen U., Baumgartner R., Bailly A., Gardiner A., Eberl L., Schulz S., Weisskopf L. (2013). Production of bioactive volatiles by different *Burkholderiaambifaria* strains. J. Chem. Ecol..

[167] Sanchez-Fernandez R.E., Diaz D., Duarte G., Lappe-Oliveras P., Sanchez S., Macias-Rubalcava M.L. (2016). Antifungal volatile organic compounds from the endophyte *Nodulisporium* sp. strain GS4d2II1a: A qualitative change in the intraspecific and interspecific interactions with *Pythium aphanidermatum*. Microorganisms.

[168] Sharma R., Chauhan A., Shirkot C.K. (2015). Characterization of plant growth-promoting *Bacillus* strains and their potential as crop protectants against *Phytophthora capsici* in tomato. Biol. Agric. Hortic..

[169] Peleg I., Feldman K. (2002). Minrav Industries Ltd.Bacillus firmus CNCM I-1582 or Bacillus cereus CNCM I-1562 for Controlling Nematodes. U.S. Patent.

[170] Susic N., Janezic S., Rupnik M., Stare B.G. (2020). Whole genome sequencing and comparative genomics of two nematicidal *Bacillus* strains reveals a wide range of possible virulence factors. G3 Genes Genomes Genet..

[171] Ruiu L., Floris I., Satta A., Ellar D.J. (2007). Toxicity of a *Brevibacilluslaterosporus* strain lacking parasporal crystals against *Musca domestica* and *Aedes aegypti*. Biol. Control.

[172] Ruiu L. (2013). *Brevibacilluslaterosporus*, a pathogen of invertebrates and a broad-spectrum antimicrobial species. Insects.

[173] Marche M.G., Camiolo S., Porceddu A., Ruiu L. (2018). Survey of *Brevibacilluslaterosporus* insecticidal protein genes and virulence factors. J. Invertebr. Pathol..

[174] Shehata M.G., Badr A.N., El Sohaimy S.A., Asker D., Awad T.S. (2019). Characterization of antifungal metabolites produced by novel lactic acid bacterium and their potential application as food biopreservatives. Ann. Agric. Sci..

[175] Wang Z., Mei X., Du M., Chen K., Jiang M., Wang K., Zalán Z., Kan J. (2020). Potential modes of action of *Pseudomonas fluorescens* ZX during biocontrol of blue mold decay on postharvest citrus. J. Sci. Food Agric..

[176] Wang Z., Zhong T., Chen K., Du M., Chen G., Chen X., Wang K., Zalán Z., Takács K., Kan J. (2021). Antifungal activity of volatile organic compounds produced by *Pseudomonas fluorescens* ZX and potential biocontrol of blue mold decay on postharvest citrus. Food Control.

[177] Blainski J.M., da Rocha Neto A.C., Schimidt E.C., Voltolini J.A., Rossi M.J., Di Piero R.M. (2018). Exopolysaccharides from *Lactobacillus plantarum* induce biochemical and physiological alterations in tomato plant against bacterial spot. Appl. Microbiol. Biotechnol..

[178] Ramadan E.M., AbdelHafez A.A., Hassan E.A., Saber F.M. (2016). Plant growth-promoting rhizobacteria and their potential for biocontrol of phytopathogens. Afr. J. Microbiol. Res..

[179] Arseneault T., Goyer C., Filion M. (2015). *Pseudomonas fluorescens* LBUM223 increases potato yield and reduces common scab symptoms in the field. Phytopathology.

[180] Fukami J., Ollero F.J., Megías M., Hungria M. (2017). Phytohormones and induction of plant-stress tolerance and defense genes by seed and foliar inoculation with *Azospirillumbrasilense* cells and metabolites promote maize growth. AMB Express.

[181] Vacheron J., Renoud S., Muller D., Babalola O.O., Prigent-Combaret C., Cassán F., Okon Y., Creus C. (2015). Alleviation of abiotic and biotic stresses in plants by *Azospirillum*. Handbook for Azospirillum, Technical Issues and Protocol.

[182] Luz C., D’Opazo V., Quiles J.M., Romano R., Mañes J., Meca G. (2020). Biopreservation of tomatoes using fermented media by lactic acid bacteria. LWT.

[183] Juodeikiene G., Bartkiene E., Cernauskas D., Cizeikiene D., Zadeike D., Lele V., Bartkevics V. (2018). Antifungal activity of lactic acid bacteria and their application for *Fusarium mycotoxin* reduction in malting wheat grains. LWT.

[184] Cao H., Meng D., Zhang W., Ye T., Yuan M., Yu J., Wu X., Li Y., Yin F., Fu C. (2021). Growth inhibition of *Fusarium graminearum* and deoxynivalenol detoxification by lactic acid bacteria and their application in sourdough bread. Int. J. Food Sci. Technol..

[185] Enebe M.C., Babaloa O.O. (2019). The impact of microbes in the orchestration of plants’ resistance to biotic stress: A disease management approach. Appl. Microbiol. Biotechnol..

[186] Kantar C., Demiray H., Dogan N.M. (2010). Role of microbial exopolymeric substances on chromium sorption and transport in heterogeneous subsurface soil. II. Binding of Cr(III) in EPS/soil system. Chemosphere.

[187] Priester J.H., Olson S.G., Webb S.M., Neu M.P., Hersman L.E., Holden P.A. (2006). Enhanced exopolymer production and chromium stabilization in *Pseudomonas putida* unsaturated biofilms. Appl. Environ. Microbiol..

[188] Kantar C., Cetin Z., Demiray H. (2008). In situ stabilization of chromium (VI) in polluted soils using organic ligands: The role of galacturonic, glucuronic and alginic acids. J. Hazard. Mater..

[189] Cetin Z., Kantar C., Alpaslan M. (2008). Interactions between uronic acids and chromium (III). Environ. Toxicol. Chem..

[190] Nair A., Juwarkar A.A., Singh S.K. (2007). Production and characterization of siderophores and its application in arsenic removal from contaminated soil. Water Air Soil Pollut..

[191] O’Brien S., Hodgson D.J., Buckling A. (2014). Social evolution of toxic metal bioremediation in *Pseudomonas aeruginosa*. Proc. R. Soc. B Biol. Sci..

[192] Rizvi A., Khan M.S. (2018). Heavy metal-induced oxidative damage and root morphology alterations of maize (*Zea mays* L.) plants and stress mitigation by metal tolerant nitrogen-fixing *Azotobacterchroococcum*. Ecotoxicol. Environ. Saf..

[193] Wang Q., Xiong D., Zhao P., Yu X., Tu B., Wang G. (2011). Effect of applying an arsenic-resistant and plant growth-promoting *Rhizobacterium* to enhance soil arsenic phyto-remediation by *Populusdeltoides* LH05-17. J. Appl. Microbiol..

[194] Gamalero E., Glick B.R. (2015). Bacterial modulation of plant ethylene levels. Plant Physiol..

[195] Vurukonda S.S.K.P., Vardharajula S., Shrivastava M., Ali S.Z. (2016). Enhancement of drought stress tolerance in crops by plant growth-promoting rhizobacteria. Microbiol. Res..

[196] Jochum M.D., McWilliams K.L., Borrego E.J., Kolomiets M.V., Niu G., Pierson E.A., Jo Y.K. (2019). Bioprospecting plant growth promoting rhizobacteria that mitigate drought stress in grasses. Front. Microbiol..

[197] Khan I., Samrah A.A., Ikram R., Rizwan M., Akhtar N., Yasmin H., Sayyed R.Z., Ali S., Ilyas N. (2020). Effects of 24-epibrassinolide on plant growth, antioxidants defense system, and endogenous hormones in two wheat varieties under drought stress. Physiol. Plant..

[198] Singh J., Singh P., Ray S., Rajput R.S., Singh H.B. (2019). Plant growth-promoting rhizobacteria: Benign and useful substitute for mitigation of biotic and abiotic stresses. Plant Growth Promoting Rhizobacteria for Sustainable Stress Management.

[199] Schobert C., Kockenberger W., Komor E. (1998). Uptake of amino acids by plants from the soil: A comparative study with castor bean seedlings grown under natural and axenic soil conditions. Plant Soil..

[200] Hayat S., Hayat Q., Alyemeni M.N., Wani A.S., Pichtel J., Ahmad A. (2012). Role of proline under changing environments. Plant Signal. Behav..

[201] Holtmann G., Bremer E. (2004). Thermoprotection of *Bacillus subtilis* by exogenously provided Glycine Betaine and structurally related compatible solutes: Involvement of Opu transporters. J. Bacteriol..

[202] Fariduddin Q., Varshney P., Yusuf M., Ali A., Ahmad A. (2013). Dissecting the role of glycine betaine in plants under abiotic stress. Plant Stress.

[203] Rahman M.S., Miyake H., Takeoka Y. (2002). Effects of exogenous glycine-betaine on growth and ultra-structure of salt-stressed rice seedlings (*Oryza sativa* L.). Plant Prod. Sci..

[204] Abdelrahman M., Jogaiah S., Burritt D.J., Tran L.P. (2018). Legume genetic resources and transcriptome dynamics under abiotic stress conditions. Plant Cell Environ..

[205] Gong M., Tang M., Chen H., Zhang Q., Feng X. (2013). Effects of two Glomus species on the growth and physiological performance of *Sophora davidii* seedlings under water stress. New For..

[206] Miglani R., Parveen N., Kumar A., Ansari M.A., Khanna S., Rawat G., Panda A.K., Bisht S.S., Upadhyay J., Ansari M.N. (2022). Degradation of xenobiotic pollutants: An environmentally sustainable approach. Metabolites.

[207] Pande V., Pandey S.C., Sati D., Bhatt P., Samant M. (2022). Microbial interventions in bioremediation of heavy metal contaminants in agroecosystem. Front. Microbiol..

[208] Datta S., Singh S., Kumar V., Dhanjal D.S., Sidhu G.K., Amin D.S., Kumar S., Singh J., Singh J. (2020). Endophytic bacteria in xenobiotic degradation. Microbial Endophytes.

[209] Bhandari S., Poudel D.K., Marahatha R., Dawadi S., Khadayat K., Phuyal S., Shrestha S., Gaire S., Basnet K., Khadka U. (2021). Microbial enzymes used in bioremediation. J. Chem..

[210] Charles S., Ratier A., Baudrot V., Multari G., Siberchicot A., Wu D., Lopes C. (2022). Taking full advantage of modelling to better assess environmental risk due to xenobiotics-the all-in-one facility MOSAIC. Environ. Sci. Pollut. Res. Int..

[211] Gangola S., Joshi S., Kumar S., Pandey S.C. (2019). Comparative analysis of fungal and bacterial enzymes in biodegradation of xenobiotic compounds. Smart Bioremediation Technologies.

[212] Sinha S., Chattopadhyay P., Pan I., Debashis B., Das K., Sen S.K. (2009). Microbial transformation of xenobiotics for environmental bioremediation. Afr. J. Biotechnol..

[213] Atashgahi S., Shetty S.A., Smidt H., De Vos W.M. (2018). Flux, impact and fate of halogenated xenobiotic compounds in the gut. Front. Physiol..

[214] Jeffries T.C., Rayu S., Nielsen U.N., Lai K., Ijaz A., Nazaries L., Singh B.K. (2018). Metagenomic Functional Potential Predicts Degradation Rates of a Model Organophosphorus Xenobiotic in Pesticide Contaminated Soils. Front. Microbiol..

[215] Davolos D., Russo F., Canfora L., Malusà E., Tartanus M., Furmanczyk E.M., Persiani A.M. (2021). A Genomic and Transcriptomic Study on the DDT-Resistant *Trichoderma hamatum* FBL 587: First Genetic Data into Mycoremediation Strategies for DDT-Polluted Sites. Microorganisms.

[216] Meng D., Zhang L., Meng J., Tian Q., Zhai L., Hao Z., Guan Z., Cai Y., Liao X. (2019). Evaluation of the Strain *Bacillus amyloliquefaciens* YP6 in Phoxim degradation via transcriptomic data and product analysis. Molecules.

[217] Dhiman N., Jasrotia T., Sharma P., Negi S., Chaudhary S., Kumar R., Mahnashi M.H., Umar A., Kumar R. (2020). Immobilization interaction between xenobiotic and *Bjerkanderaadusta* for the biodegradation of atrazine. Chemosphere.

[218] AlMatar M., Makky E.A. (2016). *Cladosporium cladosporioides* from the perspectives of medical and biotechnological approaches. 3Biotech.

[219] Esparza-Naranjo S.B., da Silva G.F., Duque-Castaño D.C., Araújo W.L., Peres C.K., Boroski M., Bonugli-Santos R.C. (2019). Potential for the biodegradation of atrazine using leaf litter fungi from a subtropical protection area. Curr. Microbiol..

[220] Al-Hawash A.B., Alkooranee J.T., Zhang X., Ma F. (2018). Fungal Degradation of Polycyclic Aromatic Hydrocarbons. Int. J. Pure Appl. Biosci..

[221] Ijoma G.N., Tekere M. (2017). Potential microbial applications of co-cultures involving ligninolytic fungi in the Bioremediation of recalcitrant xenobiotic compounds. Int. J. Environ. Sci. Technol..

[222] Kantharaj P., Boobalan B., Sooriamuthu S., Mani R. (2017). Lignocellulose Degrading Enzymes from Fungi and Their Industrial Applications. Int. J. Curr. Res. Rev..

[223] Dash D.M., Osborne W.J. (2020). Rapid biodegradation and biofilm-mediated bioremoval of organophosphorus pesticides using an indigenous *Kosakoniaoryzae* strain-VITPSCQ3 in a vertical-flow packed bed biofilm bioreactor. Ecotox. Environ. Saf..

[224] Zhou Z., Wu X., Lin Z., Pang S., Mishra S., Chen S. (2021). Biodegradation of fipronil: Current state of mechanisms of biodegradation and future perspectives. Appl. Microbiol. Biotechnol..

[225] Cairns T.C., Zheng X., Zheng P., Sun J., Meyer V. (2021). Turning inside out: Filamentous fungal secretion and its applications in biotechnology, agriculture, and the clinic. J.Fungi.

[226] Morcillo R.J.L., Baroja-Fernández E., López-Serrano L., Leal-López J., Muñoz F.J., Bahaji A., Férez-Gómez A., Pozueta-Romero J. (2022). Cell-free microbial culture filtrates as candidate biostimulants to enhance plant growth and yield and activate soil- and plant-associated beneficial microbiota. Front. Plant Sci..

[227] Feng H., Fu R., Hou X., Lv Y., Zhang N., Liu Y., Xu Z., Miao Y., Krell T., Shen Q. (2021). Chemotaxis of beneficial Rhizobacteria to root exudates: The first step towards root-microbe rhizosphere interactions. Int. J. Mol. Sci..

[228] Goswami D., Thakker J.N., Dhandhukia P.C. (2016). Portraying mechanics of plant growth-promoting rhizobacteria (PGPR): A review. Cogent Food Agric..

[229] Cretoiu M.S., Korthals G.W., Visser J.H., van Elsas J.D. (2013). Chitin amendment increases soil suppressiveness toward plant pathogens and modulates the actinobacterial and oxalobacteraceal communities in an experimental agricultural field. Appl. Environ. Microbiol..

[230] De Tender C., Mesuere B., Van der Jeugt F., Haegeman A., Ruttink T., Vandecasteele B., Dawyndt P., Debode J., Kuramae E.E. (2019). Peat substrate amended with chitin modulates the N-cycle, siderophore and chitinase responses in the lettuce rhizobiome. Sci. Rep..

[231] Sun J., Li S., Fan C., Cui K., Tan H., Qiao L., Lu L. (2022). N-Acetylglucosamine Promotes Tomato Plant Growth by Shaping the Community Structure and Metabolism of the Rhizosphere Microbiome. Microbiol. Spectr..

[232] Macias-Benitez S., Garcia-Martinez A.M., Caballero Jimenez P., Gonzalez J.M., Tejada Moral M., Parrado Rubio J. (2020). Rhizospheric Organic Acids as Biostimulants: Monitoring Feedbacks on Soil Microorganisms and Biochemical Properties. Front. Plant Sci..

[233] Acin-Albiac M., García-Jiménez B., Marín Garrido C., Borda Casas E., Velasco-Alvarez J., Serra N.S., Acedo A. (2023). Lettuce Soil Microbiome Modulated by an L-α-Amino Acid-Based Biostimulant. Agronomy.

[234] Kim D.R., Jeon C.W., Cho G., Thomashow L.S., Weller D.M., Paik M.J., Lee Y.B., Kwak Y.S. (2021). Glutamic Acid Reshapes the Plant Microbiota to Protect Plants against Pathogens. Microbiome.

[235] Colla G., Hoagland L., Ruzzi M., Cardarelli M., Bonini P., Canaguier R., Rouphael Y. (2017). Biostimulant Action of Protein Hydrolysates: Unraveling Their Effects on Plant Physiology and Microbiome. Front. Plant Sci..

[236] Luziatelli F., Ficca A.G., Colla G., BaldassarreŠvecová E., Ruzzi M. (2019). Foliar Application of Vegetal-Derived Bioactive Compounds Stimulates the Growth of Beneficial Bacteria and Enhances Microbiome Biodiversity in Lettuce. Front. Plant Sci..

[237] Ibal J.-C., Park M.-K., Park G.-S., Jung B.-K., Park T.-H., Kim M.-S., Kang G.-U., Park Y.-J., Shin J.-H. (2021). Use of Acyl-Homoserine Lactones Leads to Improved Growth of Ginseng Seedlings and Shifts in Soil Microbiome Structure. Agronomy.

[238] Spaepen S., Vanderleyden J., Remans R. (2007). Indole-3-Acetic Acid in Microbial and Microorganism-Plant Signaling. FEMS Microbiol. Rev..

[239] Duca D.R., Glick B.R. (2020). Indole-3-Acetic Acid Biosynthesis and Its Regulation in Plant-Associated Bacteria. Appl. Microbiol. Biotechnol..

[240] Eichmann R., Richards L., Schäfer P. (2021). Hormones as Go-Betweens in Plant Microbiome Assembly. Plant J..

[241] Spaepen S., Das F., Luyten E., Michiels J., Vanderleyden J. (2009). Research Letter: Indole-3-Acetic Acid-Regulated Genes in *Rhizobium etli* CNPAF512. FEMS Microbiol. Lett..

[242] Van Puyvelde S., Cloots L., Engelen K., Das F., Marchal K., Vanderleyden J., Spaepen S. (2011). Transcriptome Analysis of the Rhizosphere Bacterium *Azospirillumbrasilense* Reveals an Extensive Auxin Response. MicrobEcol.

[243] Matsukawa E., Nakagawa Y., Iimura Y., Hayakawa M. (2007). Stimulatory Effect of Indole-3-Acetic Acid on Aerial Mycelium Formation and Antibiotic Production in *Streptomyces* spp. Actinomycetologica.

[244] Laird T.S., Flores N., Leveau J.H.J. (2020). Bacterial Catabolism of Indole-3-Acetic Acid. Appl. Microbiol. Biotechnol..

[245] Rico-Jiménez M., Roca A., Krell T., Matilla M.A. (2022). A Bacterial Chemoreceptor That Mediates Chemotaxis to Two Different Plant Hormones. Environ. Microbiol..

[246] Fitzpatrick C.R., Salas-González I., Conway J.M., Finkel O.M., Gilbert S., Russ D., Teixeira P.J.P.L., Dangl J.L. (2020). The Plant Microbiome: From Ecology to Reductionism and Beyond. Annu. Rev. Microbiol..

[247] Jacoby R.P., Kopriva S. (2019). Metabolic Niches in the Rhizosphere Microbiome: New Tools and Approaches to Analyse Metabolic Mechanisms of Plant-Microbe Nutrient Exchange. J. Exp. Bot..

[248] Murillo-Roos M., Abdullah H.S.M., Debbar M., Ueberschaar N., Agler M.T. (2022). Cross-Feeding Niches among Commensal Leaf Bacteria Are Shaped by the Interaction of Strain-Level Diversity and Resource Availability. ISME J..

[249] Pascale A., Proietti S., Pantelides I.S., Stringlis I.A. (2020). Modulation of the Root Microbiome by Plant Molecules: The Basis for Targeted Disease Suppression and Plant Growth Promotion. Front. Plant Sci..

[250] Song G.C., Ryu S.Y., Kim Y.S., Lee J.Y., Choi J.S., Ryu C.M. (2013). Elicitation of Induced Resistance against *Pectobacteriumcarotovorum* and *Pseudomonas syringae* by Specific Individual Compounds Derived from Native Korean Plant Species. Molecules.

